# Persistent Luminescence in Non-Eu^2+^-Doped Compounds: A Review

**DOI:** 10.3390/ma6072789

**Published:** 2013-07-12

**Authors:** Koen Van den Eeckhout, Dirk Poelman, Philippe F. Smet

**Affiliations:** 1 LumiLab, Department of Solid State Sciences, Ghent University, Krijgslaan 281-S1, 9000 Gent, Belgium; E-Mails: koen.vandeneeckhout@ugent.be (K.V.d.E.); dirk.poelman@ugent.be (D.P.); 2 Center for Nano- and Biophotonics (NB-Photonics), Ghent University, 9000 Ghent, Belgium

**Keywords:** persistent luminescence, long-lasting phosphorescence, rare earths

## Abstract

During the past few decades, the research on persistent luminescent materials has focused mainly on Eu^2+^-doped compounds. However, the yearly number of publications on non-Eu^2+^-based materials has also increased steadily. By now, the number of known persistent phosphors has increased to over 200, of which over 80% are not based on Eu^2+^, but rather, on intrinsic host defects, transition metals (manganese, chromium, copper, *etc*.) or trivalent rare earths (cerium, terbium, dysprosium, *etc*.). In this review, we present an overview of these non-Eu^2+^-based persistent luminescent materials and their afterglow properties. We also take a closer look at some remaining challenges, such as the excitability with visible light and the possibility of energy transfer between multiple luminescent centers. Finally, we summarize the necessary elements for a complete description of a persistent luminescent material, in order to allow a more objective comparison of these phosphors.

## 1. Introduction

In most luminescent materials, the decay of the light emission lasts no longer than a few milliseconds after the end of the excitation. On the contrary, persistent phosphors can continue emitting light for minutes or hours. This phenomenon is used in safety signage, dials and displays and decoration [1], but also in less obvious applications, such as night-vision surveillance [2] or *in vivo* medical imaging [3].

**Figure 1:**
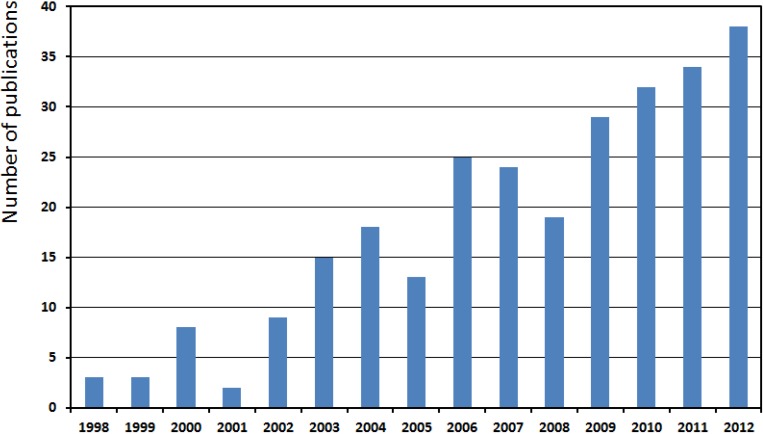
Number of papers published on non-Eu^2+^-doped persistent luminescent compounds, according to the Web of Science.

Since the discovery of SrAl_2_O_4_:Eu^2+^, Dy^3+^ in 1996 [4], many researchers and publications on persistent luminescent materials have focused on divalent europium as the activating ion. An overview of these materials has been presented in an earlier issue of this journal [5]. However, the number of publications on non-Eu^2+^-doped compounds has also seen a steady increase during the past 15 years (Figure 1). In this way, the number of materials where persistent luminescence has been observed has grown continuously over time. By now, over 200 combinations of host materials and activating ions have been described, of which less than 20% is based on divalent europium. In this review article, we will present an overview of the non-Eu^2+^-doped persistent luminescent compounds and their properties.

The research on non-Eu^2+^-based persistent luminescent materials is mainly driven by the lack of efficient red persistent phosphors. The broadband emission of Eu^2+^ is strongly dependent on the host material, more precisely, on the nephelauxetic effect (or the centroid shift) and the strength of the crystal field acting on the ion [6]. The combination of both effects leads to the so-called red shift, and the value depends strongly on the composition of the host compound and the local coordination of the europium dopant ion. It is quite common to obtain a blue or green afterglow using oxide hosts, but it is much more difficult to find a suitable host material with sufficient red shift, in order to obtain red (persistent) luminescence. Although there are a number of red emitting Eu^2+^-doped persistent phosphors, such as CaS:Eu [7,8,9] and Ca_2_Si_5_N_8_:Eu [10,11], the choice is limited and the host lattices are chemically unstable or difficult to prepare. This is especially unfortunate, since red afterglow phosphors are strongly desired for several applications, such as safety signage, paints and, more recently, also, as tracer particles for *in vivo* medical imaging [3,12,13,14]. Therefore, many research groups have focused on different luminescent ions in order to obtain an efficient red-emitting persistent phosphor.

The most obvious and popular choice for long-wavelength luminescence is Mn^2+^, known for its typical yellow-to-red emission in octahedral sites [15]. In several compounds, an energy transfer from Eu^2+^ to Mn^2+^ has been observed, leading to a red afterglow color originating from Mn^2+^, but with a long afterglow time defined by Eu^2+^. Not only red-emitting activators are being explored. Other common choices are the different trivalent rare earth ions such as Ce^3+^ and Tb^3+^. An interesting case is Dy^3+^, which shows a white emission color, due to three different emissions around 480, 575 and 665 nm. Such a white emission is very difficult to obtain with only Eu^2+^ doping. Unfortunately, these ions often require a short (UV) excitation wavelength, making it impossible to charge these persistent phosphors using visible light. Finally, several compounds are known to exhibit an afterglow without the addition of (Co) dopants, purely based on the intrinsic luminescence of the host material.

**Figure 2:**
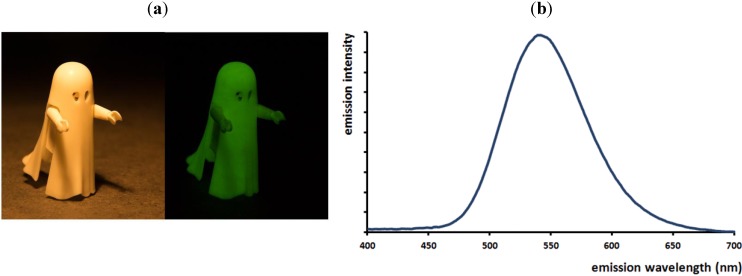
(**a**) Green persistent luminescence in a Playmobil^®^ ghost toy based on ZnS:Cu, Co. (**b**) Afterglow emission spectrum of ZnS:Cu, Co centered around 540 nm.

Until 1996, the majority of persistent luminescent applications was based on ZnS doped with copper and cobalt [4,16]. This material emits a greenish broad-band spectrum centered around 540 nm (Figure 2), which remains visible for several hours after the end of the excitation. However, the afterglow of this material is relatively weak, and it was common to add small amounts of radioactive tritium or promethium in order to sustain the luminescence [16]. Since 1996, this ZnS-based phosphor has been rendered obsolete by Eu^2+^-doped strontium and calcium aluminates exhibiting a much brighter and long-lasting afterglow. Nevertheless, the research into non-Eu^2+^-doped persistent phosphors has continuously increased in the background. An extensive list of these phosphors is presented in the following section. The most important ones (with the largest number of publications) to mention at this point are CaTiO_3_:Pr^3+^ (red), Y_2_O_2_S:Eu^3+^, Ti^4+^, Mg^2+^ (red) and CaS:Bi^3+^ (blue).

## 2. Known Compounds

This section provides an overview of the compounds where persistent luminescence, not based on divalent europium, has been reported. For every combination of host compound and activator, relevant references are indicated in the last column. In the case of energy transfer between two different dopants or luminescent centers, both the sensitizer and the activator are indicated. We use the symbol “>>” for efficient energy transfer and “>” for partial energy transfer, as derived from the emission spectra. For clarity, the materials are divided into four groups: silicates, non-silicate oxides, non-oxides and glasses. If a property was not mentioned explicitly in the text of the reference, but inferred from it or from a figure, it is put between parentheses.

Only materials with an afterglow longer than a few seconds were taken into account, since only in this case, the effect can be termed persistent luminescence. Some publications on phosphors, often using trivalent rare earth elements as dopants, claim to describe persistent luminescence, but only show an effective decay time on the order of milliseconds. In these cases, probably only the intrinsic decay of the forbidden transition within the rare earth ion is observed. Hence, these compounds and publications are deliberately not included in the tables.

The afterglow durations were taken directly from the mentioned references. However, not all of these were measured in a single, clearly defined way. The most common criterion is the visibility by the naked, dark-adapted eye. Only a few authors use the threshold value of 0.32 mcd/m^2^ (which is about 100 times the sensitivity of the human eye and a value often used in the safety signage industry [17]). In some of the references, e.g., [2,18], the afterglow duration was defined as the time the afterglow was measurable with an IR-sensitive camera (in the case of near-IR emission, one could resort to radiometric units [2]). Therefore, the afterglow durations are only noted in the tables as an indication, for a detailed comparison, we refer to the mentioned references.

Furthermore, the exact excitation conditions (wavelength, duration) are not always clear, although 254 nm is a common excitation wavelength. For details on the excitation conditions, we refer to the mentioned references.

### 2.1. Silicates

Similarly, as in Eu^2+^-doped compounds, the silicates are used as the host crystal for a large part of the non-Eu^2+^-based persistent phosphors (Table 1). Especially, the alkaline earth aluminum and magnesium silicates have been studied extensively. Some of the longest afterglow times (>5 h) have been observed in rare-earth doped CdSiO_3_, although the role of host and self-trapped exciton (STE) luminescence remains the subject of discussion in this compound [19,20].

**Table 1:** Known non-Eu^2+^-based persistent luminescent silicates (STE = self-trapped exciton).

Host material	Activators	Emission maximum (nm)	Afterglow emission	Afterglow duration	refrence
Ca_2_Al_2_SiO_7_	Ce^3+^	400–417 (blue)	identical	>1 h	[21,22,23,24]
	Ce^3+^ >> Mn^2+^	550 (yellow)	identical	>10 h	[25]
Ca_0.5_Sr_1.5_Al_2_SiO_7_	Ce^3+^ > Tb^3+^	386, 483 + 542 + 591 (white)	bluish white	>1 min	[26]
Sr_2_Al_2_SiO_7_	Ce^3+^	400 (near UV)	identical	(>2 min)	[27]
	Ce^3+^ > Dy^3+^	408, 491 + 573 (white)	(identical)	~1 h	[28]
	Ce^3+^ > Tb^3+^	410, 482 + 543 + 588 (white)	(identical)	(>1 min)	[29]
CaAl_2_Si_2_O_8_	Eu^2+^ > Mn^2+^	418, 580 (blue)	identical	>1 h	[30]
	Mn^2+^	?	?	~20 min	[31]
CaMgSi_2_O_6_	Dy^3+^	480 + 575 + 667 (white)	identical	~2 h	[32]
	Eu^2+^ > Mn^2+^	450, 580 + 680 (?)	identical	(~30 min)	[33]
	Mn^2+^	580 + 680 (red)	680 nm (red)	>1 h	[12,33,34,35,36]
SrMgSi_2_O_6_	Dy^3+^	455, 576 (blue)	identical	>5 min	[37]
	Mn^2+^	455, 612 (pink)	(identical)	(~15 min)	[37]
BaMg_2_Si_2_O_7_	Ce^3+^ > Mn^2+^	408, 680 (red)	(identical)	>2 h	[38]
	Eu^2+^ > Mn^2+^	400, 630–680 (reddish)	(identical)	>2 min	[39,40,41]
	Mn^2+^	630–680 nm (red)	(identical)	>30 min	[38,42]
Ca_2_MgSi_2_O_7_	Dy^3+^	480 + 575 + 667 (white)	identical	>3 h	[32,43]
Sr_2_MgSi_2_O_7_	Dy^3+^	441, 480 + 575 + 668 (white)	only Dy^3+^	~40 min	[44]
Ca_3_MgSi_2_O_8_	Dy^3+^	480 + 575 + 667 (white)	identical	>5 min	[32]
Sr_3_MgSi_2_O_8_	Eu^2+^ > Mn^2+^	457, 670 (?)	identical	>2 h	[45]
SrMgAl_2_SiO_7_	Ce^3+^	402 (near UV)	(identical)	>2 min	[27]
Ca_3_SnSi_2_O_9_	defects	426 (blue)	(identical)	(~10 min)	[46]
	Dy^3+^	426, 484 + 572 + 670 (white)	(identical)	(~10 min)	[46,47]
	Pr^3+^	426, 488 (greenish)	(identical)	(~10 min)	[46]
	Sm^3+^	426, 565 + 600 + 650 (red)	(identical)	(~10 min)	[46]
	Tb^3+^	426, 495 + 542 + 590 (green)	(identical)	(~10 min)	[46]
Ca_0.2_Zn_0.9_Mg_0.9_Si_2_O_6_	Eu^2+^ >> Mn^2+^	450, 580 + 680 (near IR)	identical	~1 h	[3,48]
CdSiO_3_	intrinsic/STE	380 + 467 + 560 (?)	~420 (blue)	~5 h	[19,20]
	Dy^3+^	410, 486 + 580 (white)	(identical)	>5 h	[49,50]
	Eu^3+^, Mn^2+^	587, 610 (orange)	(identical)	>1 h	[51]
	Mn^2+^	575–587 (orange)	identical	~1–5 h	[52,53,54,55]
	Mn^2+^, Tb^3+^	486 + 548, 587 (orange)	(identical)	>1 h	[56]
	Pb^2+^	498 (green)	identical	>2 h	[57]
	STE > Dy^3+^	420, 480 + 575 (white)	identical	~5 h	[20]
	STE > Eu^3+^	420, 615 (red)	identical	~5 h	[20]
	STE > Pr^3+^	420, 600 (red)	identical	~5 h	[20,58]
	STE > Sm^3+^	420, 565 + 600 (pink)	identical	~5 h	[20,59]
	STE > Tb^3+^	420, 485 + 540 (green)	identical	~5 h	[20]
	Tb^3+^	495 + 545 + 590 (green)	identical	?	[60]
Lu_2_SiO_5_	Ce^3+^	400 + 430 (blue)	(identical)	>3 h	[61,62]
MgSiO_3_	Eu^2+^ >> Mn^2+^	(456), 660–665 (?)	665 (red)	~4 h	[25,63]
Mg_2_SiO_4_	Mn^2+^	650 (red)	(identical)	~20 min	[64]
SrSiO_3_	Dy^3+^	480 + 572 + 664 (white)	identical	~1 h	[65]
Sr_2_SiO_4_	Dy^3+^	480 + 575 + 665 (white)	identical	>1 h	[66]
Sr_2_ZnSi_2_O_7_	Eu^3+^	617 (red)	identical	>20 s	[67]
Zn_2_SiO_4_	Mn^2+^	? (green)	?	(>5 min)	[68,69]
BaZrSi_3_O_9_	intrinsic/Ti^4+^	460–470 (blue)	identical	>20 s	[70,71]

### 2.2. Other Oxides

The oxides make up the majority of persistent luminescent compounds, but compared to the Eu^2+^-based materials, many more host compositions (also those in which Eu^2+^ cannot be stabilized) have been explored (Table 2). Besides the aluminates, also the stannates, titanates and germanates show some interesting properties. The longest afterglow durations have been reached in Ce^3+^-doped CaAl_4_O_7_, CaAl_2_O_4_, SrAl_2_O_4_ and BaAl_2_O_4_, all with a blue emission color. An exceptional case is the near-IR afterglow of Cr^3+^ in LiGa_5_O_8_ and Zn_3_Ga_2_Ge_2_O_10_ reported by Pan *et al.*, which could be used for night-vision surveillance or *in vivo* bio-imaging [2,18,72]. Allix *et al.* found that the latter compound is a variant of the solid solution, Zn_1+*x*_Ga_2−2*x*_Ge*_x_*O_4_:Cr^3+^, for *x* = 0.5. They report even better afterglow properties for the composition with *x* = 0.1 [73].

Pan *et al.* mention an afterglow of over 360 h (several weeks), but it should be noted that there is no agreed definition of the afterglow duration for wavelengths that cannot be detected by the human eye. This makes it difficult to compare the various reported afterglow durations.

**Table 2:** Other known non-Eu^2+^-based persistent luminescent oxides (STE = self-trapped exciton).

Host material	Activators	Emission maximum (nm)	Afterglow emission	Afterglow duration	reference
BaAl_2_O_4_	Ce^3+^	402 + 450 (blue)	(identical)	>10 h	[74]
CaAl_2_O_4_	Ce^3+^	400 (blue)	~413	>10 h	[75,76,77]
	Ce^3+^ >> Mn^2+^	525 (green)	(identical)	>10 h	[25]
	Ce^3+^ >> Tb^3+^	543 (green)	identical	>10 h	[75,76]
	Dy^3+^	477 + 491 + 577 + 668 (white)	identical	>30 min	[78]
	Eu^2+^ > Mn^2+^	440, 545 (green)	~440 (violet)	(>3 h)	[79]
	Tb^3+^	493 + 543 + 590 + 621 (green)	identical	~1 h	[75,80]
MgAl_2_O_4_	defects	520 (green)	identical	~1 h	[81]
	Cr^3+^	260, 520, 710 (?)	520, 710 (?)	(>2 h)	[82]
	Tb^3+^	?	?	~1 h	[83]
SrAl_2_O_4_	Ce^3+^	375–385 + 427 (blue)	only 385	>10 h	[84,85,86]
	Ce^3+^ > Mn^2+^	375, 515 (green)	identical	(~5 h)	[86]
	Eu^2+^ > Er^3+^	525, 1530 (green/NIR)	mainly 525	~10 min	[87]
	Eu^2+^ > Nd^3+^	515, 882 (green/NIR)	mainly 515	>15 min	[88]
CaAl_4_O_7_	Ce^3+^	325, 420 (blue)	only 420	>10 h	[84]
Sr_4_Al_14_O_25_	Ce^3+^	472 + 511 (blue/green)	(identical)	~10 min	[89]
	Eu^2+^ > Cr^3+^	490, 693 (blue/red)	mainly 490	>2 h	[90,91,92]
	Tb^3+^	542 (green)	~380 (blue)	?	[93]
Y_3_Al_5_O_12_	Ce^3+^	525 (yellow)	identical	~2 min	[94,95]
	Mn^2+^	580 (yellow-orange)	585 (orange)	~18 min	[96]
	intrinsic, Pr^3+^	300–460, 490 + 610	380, 490 + 610	?	[97]
CaYAl_3_O_7_	Ce^3+^	425 (blue)	(identical)	~min	[21]
CaO	Eu^3+^	594 + 616 (red)	(orange)	>2 h	[98,99]
	Tb^3+^	550 (green)	(identical)	?	[100]
Ga_2_O_3_	Cr^3+^	720 (near IR)	identical	>4 h	[101]
HfO_2_	intrinsic	480 (bluish white)	identical	>1 min	[102,103]
Lu_2_O_3_	Eu^3+^	611 (red)	583 + 594 + 611	>3 min	[104]
	Tb^3+^	490 + 550 (green)	identical	~5–7 h	[105,106,107,108]
SnO_2_	Sm^3+^	567 + 607 + 625 (red)	identical	~40 min	[109]
SrO	Eu^3+^	594 + 616 (orange)	identical	>1 h	[99]
	Pb^2+^	390 (violet)	identical	>1 h	[99]
	Tb^3+^	543 (green)	(identical)	?	[100]
Y_2_O_3_	Eu^3+^	612 (red)	(identical)	~90 min	[110,111]
(Zn,Mg)O	unknown	520 (orange)	(identical)	~10 min	[112,113]
ZrO_2_	Sm^3+^	570 + 614 (red)	(identical)	~15 min	[114]
	Ti^4+^ (?)	(353+) 470–500 (blue)	only 470–500	~1 h	[115,116,117,118,119]
Ba_2_SnO_4_	Sm^3+^	580 + 611 + 623 (red)	(identical)	~20 min	[120,121]
Ca_2_SnO_4_	Eu^3+^	585 + 618 + 633 (red)	(identical)	~50 min	[122,123]
	STE	410 + 466 (blue)	(identical)	~3 h	[122]
	STE >> Eu^3+^	585 + 618 + 633 (red)	(identical)	~100 min	[122]
	Sm^3+^	566 + 609 + 653 (red)	identical	>1–7 h	[120,124,125,126]
	Tb^3+^	435, 483 + 545 (blue/green)	483+545 (green)	~3 h	[127]
Mg_2_SnO_4_	intrinsic	490–495 (green)	identical	~5 h	[128,129,130]
	Mn^2+^	500 (green)	identical	>5 h	[131]
Sr_2_SnO_4_	Sb^3+^	550 (yellowish white)	identical	>2 min	[132]
	Sm^3+^	582 + 624 + 672 (red)	identical	>1 h	[120,133,134,135]
	Tb^3+^	542 (green)	(identical)	~8 min	[136]
CaSnO_3_	Pr^3+^	488 + 541 + 620 + 653 (white)	identical	>3 h	[137]
	Sm^3+^	566 + 601 + 649 + 716 (red)	(identical)	?	[138]
	Tb^3+^	491 + 545 + 588 + 622 (green)	identical	~4 h	[137,139,140]
Sr_3_Sn_2_O_7_	Sm^3+^	580 + 621 + 665 + 735 (red)	identical	>1 h	[141]
Ca_9_Gd(PO_4_)_7_	Mn^2+^	602 + 628, 660 (red)	only 660 (red)	(>20 min)	[142]
Ca_9_Lu(PO_4_)_7_	Mn^2+^	660 (red)	identical	(>20 min)	[142]
Ca_9_Tb(PO_4_)_7_	Tb^3+^	490 + 545 (green)	(identical)	(>20 min)	[142]
Ca_3_(PO_4_)_2_	Mn^2+^	645–660 (red)	identical	~1 h	[143,144]
SrMg_2_(PO_4_)_2_	Eu^3+^, Zr^4+^	500, 588(white)	(identical)	~1.5 h	[145]
SrZn_2_(PO_4_)_2_	Eu^2+^ > Mn^2+^	421, 547 (white)	(identical)	~1 min	[146]
	Mn^2+^	547 (green)	(identical)	~1 min	[146]
Zn_3_(PO_4_)_2_	Hf^4+^	470 (blue)	identical	>40 min	[147]
	Mn^2+^	616 (red)	identical	>2 h	[148,149,151]
	Mn^2+^, Zr^4−^	475, 616 (blue/red)	mainly 616	~3 h	[152]
YPO_4_	Pr^3+^	600 + 620 (orange/red)	(identical)	>30 min	[153]
Ca_0.8_Mg_0.2_TiO_3_	Pr^3+^	613 (red)	(identical)	?	[154]
CaTiO_3_	Pr^3+^	612 (red)	identical	>2 h	[155–159]
(Ca,Zn)TiO_3_	Pr^3+^	612 (red)	(identical)	~20 min	[160–162]
Ca_2_Zn_4_Ti_16_O_38_	Pr^3+^	614 + 644 (red)	mainly 614	?	[163,164]
La_2_Ti_2_O_7_	Pr^3+^	611 (red)	identical	>1 h	[165]
Gd_3_Ga_5_O_12_	Cr^3+^	697 + 716 (red)	(identical)	?	[166,167]
MgGa_2_O_4_	Mn^2+^	506 (green)	(identical)	?	[168]
LiGa_5_O_8_	Cr^3+^	716 (near IR)	identical	>1000 h	[18]
ZnGa_2_O_4_	defects	410 + 540 (white)	identical	~40 min	[169]
	Cr^3+^	650–750 (red)	identical	>1 h	[72,73]
	Mn^2+^	504 (green)	(identical)	>15 min	[170]
(Zn,Mg)Ga_2_O_4_	Mn^2+^	505 (green)	(identical)	>15 min	[170]
Cd_2_Ge_7_O_16_	Mn^2+^	585 (orange)	identical	>3 h	[171]
	Pb^2+^	352 + 497 (blue)	only 497	~10 min	[172]
MgGeO_3_	Mn^2+^	650–670 (red)	identical	~30 min	[173,174]
Zn_2_GeO_4_	Mn^2+^	528 (green)	(identical)	>2 h	[175]
CaZnGe_2_O_6_	Dy^3+^	(white)	(identical)	>3 h	[176]
	Mn^2+^	648 (red)	identical	>3 h	[177]
	Tb^3+^	488 + 552 + 583 + 622 (green)	identical	~4 h	[178,179]
Cd_3_Al_2_Ge_3_O_12_	intrinsic > Dy^3+^	437, 485 + 580 (?)	(identical)	~1 h	[180]
La_3_Ga_5_GeO_14_	Cr^3+^	785, 960–1030 (near IR)	only 960–1030	>1–8 h	[181,182]
Zn_3_Ga_2_Ge_2_O_10_	Cr^3+^	696 + 713 (near IR)	identical	>360 h	[2,73]
CaMoO_4_	Eu^3+^	616 (red)	identical	>5 min	[183]
NaNbO_3_	Pr^3+^	620 (red)	identical	?	[184]
YTaO_4_	Tb^3+^	492 + 543 + 590 + 624 (green)	(identical)	~2 h	[185]
CaWO_4_	intrinsic > Pr^3+^	415, 490 + 650 (blue/white)	identical	>10 min	[186]
	Eu^3+^	592 + 616 (red)	identical	~40 min	[187,188,189]
	Sm^3+^ >> Eu^3+^	592 + 616 (red)	(identical)	>35 min	[190]
	Tb^3+^	490 + 546 (green)	identical	(>10 min)	[191]
BaZrO_3_	defects (F_A_)	408 (blue)	identical	~30 min	[192]
	Eu^3+^	574 + 596 + 614 (red)	(identical)	(~10 min)	[192]
	Ti >> Eu^3+^	574 + 596 + 614 (red)	(identical)	(~10 min)	[193]

### 2.3. Other Compounds

The sulfides (Table 3) have the longest recorded history of all persistent luminescent compounds. In fact, the famous Bologna Stone, discovered by Vincenzo Casciarolo in 1602 [194], consisted mainly of copper-doped BaS [195]. Nowadays, the use of ZnS: Cu has much decreased in favor of SrAl_2_O_4_:Eu, Dy. The focus has mainly shifted to the oxysulfides, especially Y_2_O_2_S:Eu^3+^, Ti^4+^, Mg^2+^, which is currently one of the best red-emitting persistent phosphors. Nevertheless, its afterglow intensity is much weaker than the Eu^2+^-doped aluminates or silicates [196]. An interesting case of persistent luminescence is observed in undoped BCNO, where the emission wavelength can be shifted from blue to orange purely by changing the preparation conditions.

**Table 3:** Other known non-Eu^2+^-based persistent luminescent compounds.

Host material	Activators	Emission maximum (nm)	Afterglow emission	Afterglow duration	reference
BaS	Cu^+^	610 (orange)	(identical)	>30 min	[195]
CaS	Bi^3+^	448 (blue)	(identical)	(~20 min)	[8,197,198,199]
	Ce^3+^	508 + 568 (green)	(identical)	~5 min	[200]
	Sm^3+^	569 (green)	?	(~3 h)	[201]
(Ca,Sr)S	Bi^3+^	453 (blue)	(identical)	(>15 min)	[202]
SrS	defects	517 (green)	(identical)	(~20 min)	[203]
ZnS	Cu^+^	530 (green)	(identical)	(>3 h)	[16,69,204,205]
Gd_2_O_2_S	Ti^3+^/defects	590 (orange)	identical	~2 h	[206,207]
	Ti^3+^ > Er^3+^	555 + 675 (green)	555 + 675, 590	>1 h	[206,208]
	Ti^3+^ >> Eu^3+^	504 + 536 + 620 (red)	identical	(>5 min)	[206,209,210]
	Ti > Sm^3+^	607 (red)	590, 607	?	[206]
	Ti > Tm^3+^	513 + 800 (?)	590, 800	?	[206]
La_2_O_2_S	Sm^3+^	605 + 645 + 656 (red)	(identical)	(>1 min)	[211]
Y_2_O_2_S	Ti^3+^/defects	540–594 (orange)	identical	>5 h	[212,213,214,215,216]
	Eu^3+^	590 + 614 + 627 + 710 (red)	identical	~3 h	[217,218,219,220]
	Sm^3+^	570 + 606 + 659 (red)	(identical)	>1 h	[221,222,223]
	Tb^3+^	417 + 546 (green)	(identical)	>20 min	[224]
	Ti^3+^ > Eu^3+^	616 + 625 (red)	565, 616 + 625	~10 min–5 h	[225,226]
	Tm^3+^	495 + 545 + 588 (orange)	identical	~1 h	[227]
BCNO	intrinsic	520 (green)	identical	>2 h	[228,229]
Ba_5_(PO_4_)_3_Cl	Ce^3+^ >> Eu^2+^	350, 435 (blue)	only 435	(>5 min)	[230]
KY_3_F_10_	Sm^3+^	558 + 597 + 651 (red)	(identical)	(>2 min)	[231]
ZnSiN_2_	Mn^2+^	620 (red)	(identical)	~min	[232]

### 2.4. Glasses

A final group of persistent luminescent compounds are the glasses (Table 4). Although it is sometimes difficult to accurately infer the composition of these glasses from the publications, some clear trends can be observed. Especially, the calcium aluminum silicate and zinc boron silicate glasses have a long afterglow of more than one hour.

**Table 4:** Known non-Eu^2+^-based persistent luminescent glasses.

Host material	Activators	Emission maximum (nm)	Afterglow emission	Afterglow duration	reference
Ca_4_Al_6_Si_3_O_19_	Ce^3+^	? (blue)	(identical)	>1 h	[233]
	Pr^3+^	? (red)	(identical)	>1 h	[233]
	Tb^3+^	350–600 (green)	identical	>1 h	[233]
Ca_59_Al_54_Si_7_Mg_7_O_161_	Mn^2+^	540 (yellow)	(identical)	>1 h	[234]
	Pr^3+^	493 + 610 (red)	(identical)	>1 h	[234]
	Tb^3+^	543 (green)	(identical)	>2 h	[234,235,236]
GeO_2_	intrinsic	465 (blue)	identical	(~20 min)	[237,238]
SiO_2_	defects	290 + 390 (blue)	identical	~1 h	[239]
Na_2_AlB_15_O_25_	Mn^2+^	590 (reddish)	identical	~5 min	[240]
Na_4_CaGa_8_Si_3_O_21_	Tb^3+^	542 (green)	identical	~1 h	[241]
Na_4_CaSi_7_O_17_	Cu^+^/Cu^2+^	510 (blue green)	identical	>30 min	[242]
Sr_7_B_26_O_46_	Eu^2+^, Ce^3+^	350, 430 (blue)	mainly 430	(>2 min)	[243]
ZnGe_3_O_7_	Mn^2+^	534 (green)	identical	>1 h	[244]
Zn_2_GeO_4_	Mn^2+^	540 (green)	identical	(>10 s)	[245]
Zn_3_B_2_SiO_8_	Pr^3+^	495 + 603 (reddish)	identical	(>30 min)	[246]
	Tb^3+^	542 (green)	identical	~1 h	[247,248]
Zn_11_B_8_Si_5_O_33_	Mn^2+^	525–606 (green/yellow)	identical	~12 h	[249]
Zn_11_B_10_Si_4_O_34_	Mn^2+^	590 (red)	identical	(~20 min)	[250]
	Mn^2+^, Sm^3+^	600 (red)	identical	~10 h	[251,252]
	Mn^2+^, Yb^3+^	605, 980 (red/IR)	identical	(~10 min)	[253]
Zn_60_B_40_Si_17_Ge_3_A_l4_O_160_	defects	410 (blue)	identical	~2 h	[254]

## 3. General Remarks

**Figure 3:**
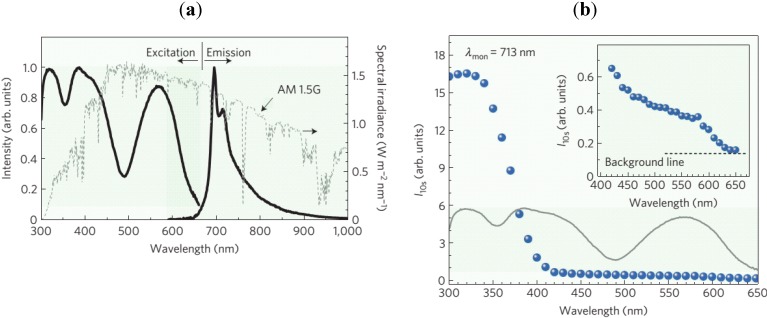
(**a**) Excitation and emission spectrum of Zn_3_Ga_2_Ge_2_O_10_:0.5%Cr^3+^; (**b**) Effectiveness of excitation wavelength (energy) for persistent luminescence of Zn_3_Ga_2_Ge_2_O_10_:0.5%Cr^3+^. The afterglow intensity after 10 s is monitored as a function of the excitation wavelength (Reprinted with permission from [2]).

It is very difficult to draw general conclusions from the above tables. One of the most interesting activators is Cr^3+^, which is not commonly used, but shows some excellent afterglow properties as a red/near-IR luminescent center. This might be especially useful for *in vivo* medical imaging applications. Unfortunately, even though the excitation spectrum of Cr^3+^ for steady-state luminescence extends to about 650 nm, it is very difficult to fill the traps, which are necessary to obtain afterglow, using visible light (about 40 times less efficient compared to UV light) [2] (Figure 3).

### 3.1. Excitation Difficulties

**Figure 4:**
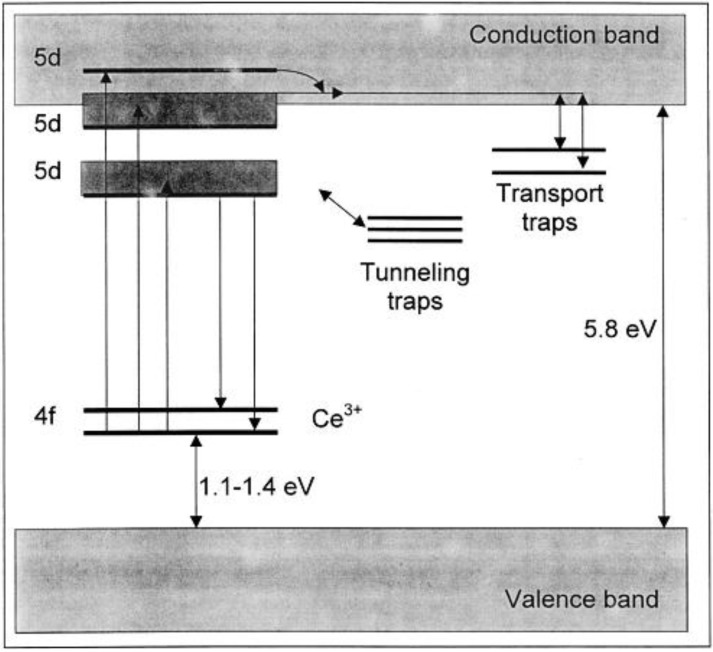
Energy level diagram for CaAl_2_O_4_: Ce^3+^, showing the positions of the Ce^3+^ levels relative to the bandgap of the host and the proposed trapping mechanism. After excitation in the conduction band, trapping occurs through the conduction band. After excitation in the lower 5d levels, trapping occurs through tunneling (Reprinted with permission from [77]. Copyright 2003 The Electrochemical Society).

From Figure 3, it is immediately clear that the steady-state excitation spectrum and afterglow excitation spectrum are not always the same. In many persistent luminescent materials, it is much easier to fill traps using higher energy photons (*i.e*., using shorter excitation wavelengths) [2,255]. This implies that direct bandgap excitation is much more efficient to fill the traps than excitation of the luminescent centers. Even more problematic, the latter type of excitation might require a certain thermal activation barrier to be surpassed before traps can be filled [255], making the use of visible light even less favorable. Of course, it is also possible to fill the traps directly through tunneling from the activating ions, which does not require short wavelength excitation, but is clearly less efficient. These different trapping processes are shown on an energy level diagram in Figure 4.

This effect appears to be even more profound in non-Eu^2+^-based persistent phosphors, where, in general, only UV light is able to effectively fill the traps in the material. This implies that the role of the host compound is much larger than in Eu^2+^-based materials. While it has been shown that in Eu^2+^-based persistent phosphors, the activator is a main source of trapped electrons [256], in non-Eu^2+^-based compounds, the trapped charge carriers are created mainly after band gap excitation. The luminescent center is subsequently excited by energy transferred from the traps when the trapped electron and hole recombine. The same phenomenon is illustrated by the fact that the afterglow duration is influenced much more by the host compound than by the actual luminescent center. Indeed, by looking at the tables presented in Section 2, it is not uncommon to see certain host compounds with very similar afterglow durations irrespective of the activator being, e.g., Pr^3+^, Sm^3+^ or Tb^3+^.

The fact that UV excitation is required for efficient trap filling is especially unfortunate for persistent phosphors based on Dy^3+^. This could be an excellent activator for white persistent luminescence, e.g., in paints, signage and displays. However, since indoor lighting contains little to no UV wavelengths (especially with the advent of LED lighting [257]), these compounds are not suited for practical indoor applications.

### 3.2. Energy Transfer

In several persistent luminescent compounds, energy transfer has been reported. Two types of energy transfer can be distinguished in this case. The first type is the transfer of excitation energy between a sensitizer and an activator. However, we are more interested in the second type, where energy is transferred during the afterglow phase, after the end of the excitation. When the first activating ion recombines, instead of emitting a photon, it can transfer this recombination energy to a second activating ion. This makes it possible to see or extend the afterglow emission from activators that usually have little to no persistent luminescent properties. If the energy transfer is very efficient, only emission from the second activator, receiving the recombination energy, can be observed. In the other case, luminescence from both kinds of activators can be seen simultaneously in the afterglow spectrum.

It is not always immediately clear if energy transfer is present or not. The afterglow spectrum can consist of the emission of two different kinds of activators, even when no energy is transferred between them. It is therefore necessary to carefully inspect the decay behavior of both kinds of activators. If the decay rates of both are the same, this indicates that one of them is transferring its recombination energy to the other. If no energy transfer is present, it is likely that both kinds of activators will have a (slightly) different decay behavior, and the shape of the afterglow spectrum might change over time.

## 4. Tools for an Accurate Description of a Persistent Luminescent Material

There is no standard way to describe the properties of a given persistent luminescent material. The multitude of parameters, the uncertainties about the underlying mechanism and the lack of clear definitions make an accurate and complete description or comparison nearly impossible. Ideally, there are certain elements and experiments that should always be addressed in a publication on persistent phosphors. This allows for an easier interpretation of experimental results and simplifies the comparison between different persistent luminescent materials.

The emission and excitation spectrum during fluorescence should be given, in order to know which activators are taking part in the luminescent process. When multiple peaks or bands are present in the excitation or emission spectrum, the corresponding emission and excitation spectra for each peak should be measured. Ideally, an excitation-emission mapping is provided, offering a complete overview of the emission spectrum for every possible excitation wavelength. This can also unveil the presence of energy transfer during the excitation or emission process.

**Figure 5:**
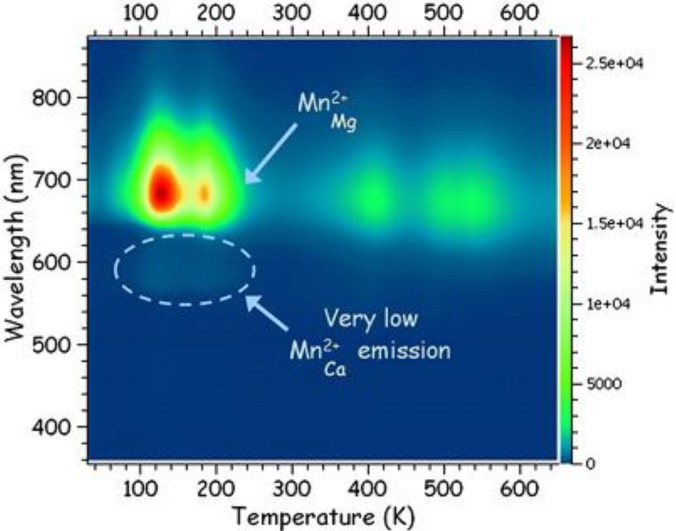
Thermoluminescence (TL)-emission mapping: the emission spectrum is monitored during the thermoluminescence experiment, showing which traps are related to which activators. An example is shown for Mn^2+^-emission in CaMgSi_2_O_6_ (Reprinted with permission from [33]. Copyright 2010 Elsevier)

Not only the steady-state emission spectrum during excitation, but also the afterglow emission spectrum should be shown, since these can differ drastically from each other. In this way, it is clear which activators are taking part in the persistent luminescence and which don’t. If the afterglow emission spectrum changes over time, it might be valuable to show the spectrum at different time intervals after the excitation. For applications, it might be useful to mention both the fluorescence and the afterglow color. If multiple luminescent centers are present, a thermoluminescence (TL) experiment, where the emission spectrum is measured (TL-emission mapping, Figure 5), provides information about which traps are connected to which luminescent centers. For example, a certain activator might only emit at higher temperatures, indicating that it is only connected to deeper traps in the material.

**Figure 6:**
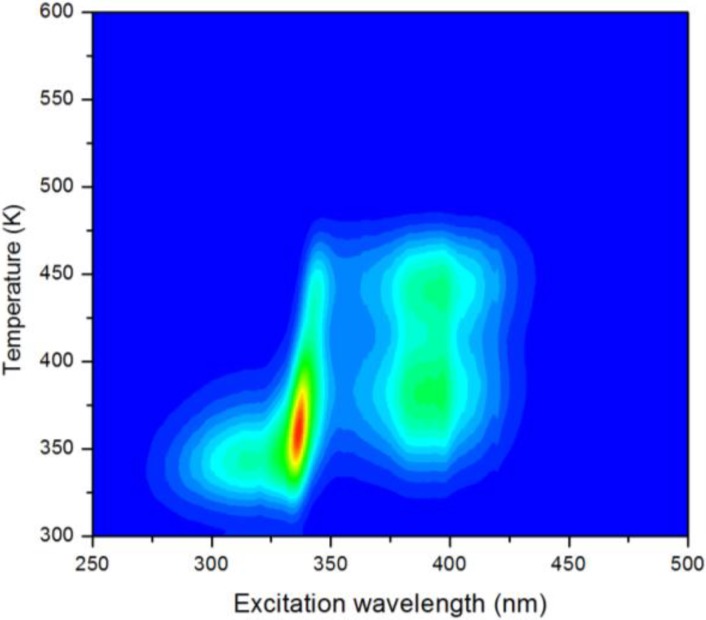
TL-excitation mapping: the TL measurement is repeated for different excitation wavelengths, showing which wavelengths are suited for trap filling. An example is shown for Cu^+^-emission in ZnS (presented earlier in [258]). It can be seen that different kinds of traps are being filled by short (<340 nm) and longer (>340 nm) wavelengths, where 340 nm corresponds to the band gap of the ZnS host compound.

If any TL measurements are made, it is advisable to perform an entire series instead of a single experiment, by varying a single parameter and keeping the other parameters constant. These parameters include the duration of the excitation, the excitation intensity, the heating rate and the delay between excitation and the start of the TL experiment (fading time). A TL-excitation mapping (Figure 6)—where the TL experiment is repeated for various excitation wavelengths—is especially useful, since it directly provides information on the trap filling probability of different wavelengths [258]; *i.e*., it shows which excitation wavelengths are suitable for inducing persistent luminescence. This can then be compared to the steady-state excitation spectrum to see which processes occur during fluorescence and during trap filling.

A further study of the trapping system can be done by performing TL experiments after excitation at different temperatures or by partial thermal emptying of the sample traps before the experiment. In this way, the depth of the various traps can be obtained or the presence of a trap depth distribution can be revealed [259]. Indeed, if such a distribution is present, exciting at higher temperatures or partial thermal cleaning will lead to only deeper traps being filled and shallower traps being emptied. Therefore, the estimated trap depth obtained from a TL experiment will become continuously deeper for higher excitation temperatures, which proves the existence of the trap depth distribution. Under the right conditions, it is even possible to derive the shape of this distribution (Gaussian, uniform, exponential, *etc*.) [260].

For further study of the defects, one can also turn to electron paramagnetic/spin resonance (EPR/ESR), which provides more information on the structural properties of the defects [24,261].

Finally, it is important to clearly state the exact experimental conditions, such as the dopant and codopant concentrations and the excitation wavelength and duration. If the duration of the afterglow decay is given, information should be given on how this was determined. According to DIN 67510-1, the sample should be excited for five minutes by 1000 lx light of an unfiltered Xe arc lamp. However, the emission spectrum of a Xe lamp is very broad and contains UV, visible, as well as infrared light. This makes it hard to draw conclusions on the excitability, based on such a measurement. It does not give a good prediction of how the persistent luminescent material will behave when excited by artificial light or sunlight. It might be more interesting to excite with monochromatic light at different wavelength and compare the afterglow in each situation.

The afterglow intensity decay should be measured in cd/m^2^, and the afterglow duration should be the time between the end of the excitation and the moment when the afterglow intensity drops below 0.32 mcd/m^2^, a value commonly used by the safety signage industry (about 100-times the sensitivity of the dark-adapted eye [17]). In this way, it would be very simple and straightforward to compare the performance of different persistent luminescent materials. However, this definition is not applicable for UV- or NIR-emitting persistent phosphors, where the luminous emission is zero by default and no clear definition exists for the afterglow duration. In that case, one can resort to radiometric units [2].

Furthermore, such absolute measurements of the afterglow decay could provide important information on the absolute concentration of activators, defects and trapped charge carriers.

## 5. Conclusions and Perspectives

A lot of research is going on in the field of non-Eu^2+^ persistent luminescent materials, and numerous material-dopant combinations have been and are being developed. However, up to now, the best Eu^2+^-based persistent phosphors are still without competition in terms of absolute luminance and afterglow time, apart from certain Cr^3+^-doped phosphors. Since the process of persistent luminescence is based on a delicate interplay between energy levels of dopants and co-dopants, intrinsic defects and energy bands of the host lattice and the possible physical proximity of dopants and co-dopants, small changes in composition, material purity and crystallinity and dopant concentration can have a strong effect on the afterglow properties. Most probably, the optimum material properties, especially the total amount of stored energy, have not been achieved yet, and most likely, some of the persistent phosphors listed in the tables of this review still have to show their real potential to shine.

While Eu^2+^-doped persistent phosphors are still unrivalled for blue and green emission, the use of other dopants allows one to extend the wavelength range that can be covered with persistent luminescence. Probably, the potential applications, especially in the red and near-infrared range, will be a driving force into further research and developments of new non-Eu^2+^-based materials.

In order to be able to compare experimental research obtained by different research groups on identical or different phosphor compositions, there is an urgent need for a more standardized way of measuring and defining persistent phosphor properties. The standard for measuring light output in cd/m^2^ is questionable, since the eye sensitivity shifts to shorter wavelengths at lower light levels [196,262,263]. In addition, the standard way of exciting persistent phosphors, using an unfiltered Xe-arc, containing large amounts of short wavelength ultraviolet radiation, is not a realistic approach and cannot be compared to solar or artificial indoor illumination. Finally, a new standard is needed for quantifying the performance of ultraviolet or infrared emitters: since the eye sensitivity is zero at these wavelengths, photopic units cannot be used and performance should be quoted in radiometric units or numbers of photons.

## References

[1] Smet P.F., Poelman D., Hehlen M.P. (2012). Focus Issue Introduction: Persistent Phosphors. Opt. Mater. Express.

[2] Pan Z., Lu Y.Y., Liu F. (2012). Sunlight-Activated Long-Persistent Luminescence in the Near-Infrared from Cr^3+^-Doped Zinc Gallogermanates. Nat. Mater..

[3] Le Masne de Chermont Q., Chanéac C., Seguin J., Pellé F., Maîtrejean S., Jolivet J.P., Gourier D., Bessodes M., Scherman D. (2007). Nanoprobes with Near-Infrared Persistent Luminescence for *in vivo* Imaging. Proc. Natl. Acad. Sci. USA.

[4] Matsuzawa T., Aoki Y., Takeuchi N., Murayama Y. (1996). A New Long Phosphorescent Phosphor with High Brightness, SrAl_2_O_4_:Eu^2+^, Dy^3+^. J. Electrochem. Soc..

[5] Van den Eeckhout K., Smet P.F., Poelman D. (2010). Persistent Luminescence in Eu^2+^-Doped Compounds: A Review. Mater..

[6] Dorenbos P. (2003). Energy of the First 4f^7^→4f^6^5d Transition of Eu^2+^ in Inorganic Compounds. J. Lumin..

[7] Jia D., Jia W., Evans D.R., Dennis W.M., Liu H., Zhu J., Yen W.M. (2000). Trapping Processes in CaS:Eu^2+^, Tm^3+^. J. Appl. Phys..

[8] Jia D., Zhu J., Wu B. (2000). Trapping Centers in CaS:Bi^3+^ and CaS:Eu^2+^, Tm^3+^. J. Electrochem. Soc..

[9] Jia D. (2006). Enhancement of Long-Persistence by Ce Co-Doping in CaS:Eu^2+^, Tm^3+^ Red Phosphor. J. Electrochem. Soc..

[10] Miyamoto Y., Kato H., Honna Y., Yamamoto H., Ohmi K. (2009). An Orange-Emitting, Long-Persistent Phosphor, Ca_2_Si_5_N_8_:Eu^2+^, Tm^3+^. J. Electrochem. Soc..

[11] Van den Eeckhout K., Smet P.F., Poelman D. (2009). Persistent Luminescence in Rare-Earth Codoped Ca_2_Si_5_N_8_:Eu^2+^. J. Lumin..

[12] Maldiney T., Lecointre A., Viana B., Bessière A., Bessodes M., Gourier D., Richard C., Scherman D. (2011). Controlling Electron Trap Depth to Enhance Optical Properties of Persistent Luminescence Nanoparticles for *in vivo* Imaging. J. Am. Chem. Soc..

[13] Maldiney T., Richard C., Seguin J., Wattier N., Bessodes M., Scherman D. (2011). Effect of Core Diameter, Surface Coating, and PEG Chain Length on the Biodistribution of Persistent Luminescence Nanoparticles in Mice. ACS Nano.

[14] Maldiney T., Sraiki G., Viana B., Gourier D., Richard C., Scherman D., Bessodes M., Van den Eeckhout K., Poelman D., Smet P.F. (2012). *In vivo* Optical Imaging with Rare Earth Doped Ca_2_Si_5_N_8_ Persistent Luminescence Nanoparticles. Opt. Mater. Express.

[15] Yen W.M., Shionoya S., Yamamoto H. (2007). Section 3.2.5, Mn^2+^ Phosphors (3d^5^). Phosphor Handbook.

[16] Yen W.M., Shionoya S., Yamamoto H. (2007). Section 12.3.1, History of Long Persistent Phosphors. Phosphor Handbook.

[17] Clabau F., Rocquefelte X., Jobic S., Deniard P., Whangbo M.H., Garcia A., Le Mercier T. (2007). On the Phosphorescence Mechanism in SrAl_2_O_4_:Eu^2+^ and Its Codoped Derivatives. Solid State Sci..

[18] Liu F., Yan W., Chuang Y.J., Zhen Z., Xie J., Pan Z. (2013). Photostimulated Near-Infrared Persistent Luminescence as a New Optical Read-Out from Cr3^+^-Doped LiGa_5_O_8_. Sci. Rep..

[19] Kuang J.Y., Liu Y.L. (2006). Trapping effects in CdSiO_3_:In^3+^ Long Afterglow Phosphor. Chin. Phys. Lett..

[20] Liu Y., Kuang J., Lei B., Shi C. (2005). Color-Control of Long-Lasting Phosphorescence (LLP) Through Rare Earth Ion-Doped Cadmium Metasilicate Phosphors. J. Mater. Chem..

[21] Kodama N., Takahashi T., Yamaga M., Tanii Y., Qiu J., Hirao K. (1999). Long-Lasting Phosphorescence in Ce^3+^-Doped Ca_2_Al_2_SiO_7_ and CaYAl_3_O_7_ Crystals. Appl. Phys. Lett..

[22] Kodama N., Tanii Y., Yamaga M. (2000). Optical Properties of Long-Lasting Phosphorescent Crystals Ce^3+^-Doped Ca_2_Al_2_SiO_7_ and CaYAl_3_O_7_. J. Lumin..

[23] Wu H., Hu Y., Ju G., Chen L., Wang X., Yang Z. (2011). Photoluminescence and Thermoluminescence of Ce^3+^ and Eu^2+^ in Ca_2_Al_2_SiO_7_ Matrix. J. Lumin..

[24] Yamaga M., Tanii Y., Kodama N., Takahashi T., Honda M. (2002). Mechanism of Long-Lasting Phosphorescence Process of Ce^3+^-Doped Ca_2_Al_2_SiO_7_ Melilite Crystals. Phys. Rev. B.

[25] Wang X.J., Jia D., Yen W.M. (2003). Mn^2+^ Activated Green, Yellow, and Red Long Persistent Phosphors. J. Lumin..

[26] Ito Y., Komeno A., Uematsu K., Toda K., Sato M. (2006). Luminescence Properties of Long-Persistence Silicate Phosphors. J. Alloy. Compd..

[27] Gutiérrez-Martín F., Fernández-Martinez F., Díaz P., Colón C., Alonso-Medina A. (2010). Persistent UV Phosphors for Application in Photo catalysis. J. Alloys Compd..

[28] Gong Y., Wang Y., Li Y., Xu X. (2010). Ce^3+^, Dy^3+^ Co-Doped White-Light Long-Lasting Phosphor: Sr_2_Al_2_SiO_7_ Through Energy Transfer. J. Electrochem. Soc..

[29] Pan W., Ning G., Lin Y., Yang X. (2008). Sol-Gel Processed Ce^3+^, Tb^3+^ Codoped White Emitting Phosphors in Sr_2_Al_2_SiO_7_. J. Rare Earths.

[30] Zhang J., Chen B., Sun J., Li X., Cheng L., Zhong H. (2012). White Long-Lasting Phosphorescence Generation in a CaAl_2_Si_2_O_8_:Eu^2+^, Mn^2+^, Dy^3+^ System Through Persistent Energy Transfer. J. Phys. D: Appl. Phys..

[31] Chen B.S., Zheng Z.S., Lin Y.M., Chen G.L., Zhou L., Guo H.X., Huang L.F. (2011). Preparation of a Novel Red Long Lasting Phosphorescent Material CaAl_2_Si_2_O_8_:Mn^2+^ and Investigation of Its Luminescent Properties. Appl. Mech. Mater..

[32] Chen Y., Cheng X., Liu M., Qi Z., Shi C. (2009). Comparison Study of the Luminescent Properties of the White-Light Long Afterglow Phosphors: Ca*_x_*MgSi_2_O_5+*x*_:Dy^3+^ (*x* = 1, 2, 3). J. Lumin..

[33] Lecointre A., Bessière A., Viana B., Gourier D. (2010). Red Persistent Luminescent Silicate Nanoparticles. Radiat. Meas..

[34] Bessière A., Lecointre A., Priolkar K.R., Gourier D. (2012). Role of Crystal Defects in Red Long-Lasting Phosphorescence of CaMgSi_2_O_6_:Mn Diopsides. J. Mater. Chem..

[35] Maldiney T., Lecointre A., Viana B., Bessiere A., Gourier D., Bessodes M., Richard C., Scherman D. Trap Depth Optimization to Improve Optical Properties of Diopside-Based Nanophosphors for Medical Imaging. Proceedings of Oxide-Based Materials and Devices III.

[36] Lecointre A., Bessière A., Priolkar K.R., Gourier D., Wallez G., Viana B. (2013). Role of Manganese in Red Long-Lasting Phosphorescence of Manganese-Doped Diopside for *in vivo* Imaging. Mater. Res. Bull..

[37] He Z., Wang X.J., Yen W.M. (2007). Behavior of Mn^2+^ Ions in the Trapping Process of SrMg(SiO_3_)_2_:Mn, Dy. J. Lumin..

[38] Gong Y., Xu X.H., Zeng W., Wu C.J., Wang Y.H. (2012). Ce^3+^, Mn^2+^ Co-Doped Red-Light Long-Lasting Phosphor: BaMg_2_Si_2_O_7_ Through Energy Transfer. Phys. Proced..

[39] Abe S., Uematsu K., Toda K., Sato M. (2006). Luminescent Properties of Red Long Persistence Phosphors, BaMg_2_Si_2_O_7_:Eu^2+^, Mn^2+^. J. Alloys Compd..

[40] Aitasalo T., Hietikko A., Hreniak D., Hölsä J., Lastusaari M., Niittykoski J., Stręk W. (2008). Luminescence Properties of BaMg_2_Si_2_O_7_:Eu^2+^, Mn^2+^. J. Alloys Compd..

[41] Ye S., Zhang J., Zhang X., Lu S., Ren X., Wang X.J. (2007). Mn^2+^ Activated Red Phosphorescence in BaMg_2_Si_2_O_7_:Mn^2+^, Eu^2+^, Dy^3+^ Through Persistent Energy Transfer. J. Appl. Phys..

[42] Ye S., Zhang J., Zhang X., Wang X. (2007). Mn^2+^ Activated Red Long Persistent Phosphors in BaMg_2_Si_2_O_7_. J. Lumin..

[43] Lin L., Zhao Z., Zhang W., Zheng Z., Yin M. (2009). Photo-Luminescence Properties and Thermo-Luminescence Curve Analysis of a New White Long-Lasting Phosphor:Ca_2_MgSi_2_O_7_:Dy^3+^. J. Rare Earths.

[44] Liu B., Kong L., Shi C. (2007). White-Light Long-Lasting Phosphor Sr_2_MgSi_2_O_7_:Dy^3+^. J. Lumin..

[45] Gong Y., Wang Y., Xu X., Li Y., Xin S., Shi L. (2011). The Persistent Energy Transfer of Eu^2+^ and Mn^2+^ and the Thermoluminescence Properties of Long-Lasting Phosphor Sr_3_MgSi_2_O_8_:Eu^2+^, Mn^2+^, Dy^3+^. Opt. Mater..

[46] Xu X., Wang Y., Zeng W., Gong Y., Liu B. (2011). Luminescent Properties of the Multicolor Afterglow Phosphors Ca_3_SnSi_2_O_9_:Re^3+^ (Re = Pr, Tb, Sm). J. Am. Ceram. Soc..

[47] Wei R.P., Ju Z.H., Ma J.X., Zhang D., Zang Z.P., Liu W.S. (2009). A Novel White Afterglow Phosphorescent Phosphor Ca_3_SnSi_2_O_9_:Dy^3+^. J. Alloys Compd..

[48] Lecointre A., Viana B., LeMasne Q., Bessière A., Chanéac C., Gourier D. (2009). Red Long-Lasting Luminescence in Clinoenstatite. J. Lumin..

[49] Lei B.F., Liu Y.L., Ye Z.R., Shi C.S. (2004). A Novel White Light Emitting Long-Lasting Phosphor. Chin. Chem. Lett..

[50] Liu Y.L., Lei B., Shi C.H. (2005). Luminescent Properties of a White Afterglow Phosphor CdSiO_3_:Dy^3+^. Chem. Mater..

[51] Qu X., Cao L., Liu W., Su G., Wang P. (2009). Luminescence Properties of CdSiO_3_:Mn^2+^, RE^3+^ (RE = Sm, Dy, Eu) Phosphors. J. Alloys Compd..

[52] Kuang J., Liu Y., Lei B. (2006). Effect of RE^3+^ as a Co-Dopant in Long-Lasting Phosphorescence CdSiO_3_:Mn^2+^ (RE = Y, La, Gd, Lu). J. Lumin..

[53] Lei B., Liu Y., Ye Z., Shi C. (2004). Luminescence Properties of CdSiO_3_:Mn^2+^ Phosphor. J. Lumin..

[54] Qu X., Cao L., Liu W., Su G., Xu C., Wang P. (2010). Preparation and Properties of CdSiO_3_:Mn^2+^, Dy^3+^ Phosphor. J. Alloys Compd..

[55] Qu X.F., Cao L.X., Liu W., Su G. (2012). Sol-Gel Synthesis and Luminescence Properties of CdSiO_3_: Mn^2+^, Eu^3+^ Phosphor. J. Alloys Compd..

[56] Qu X., Cao L., Liu W., Su G. (2012). Preparation and Properties of CdSiO_3_:Mn^2+^, Tb^3+^ Phosphor. Ceram. Int..

[57] Kuang J., Liu Y. (2006). Luminescence Properties of a Pb^2+^ Activated Long-Afterglow Phosphor. J. Electrochem. Soc..

[58] Kuang J., Liu Y. (2006). Observation of Energy Transfer from Host to Rare Earth Ions in Pr^3+^-Doped CdSiO_3_ Long-Lasting Phosphor. Chem. Phys. Lett..

[59] Lei B., Liu Y., Liu J., Ye Z., Shi C. (2004). Pink Light Emitting Long-Lasting Phosphorescence in Sm^3+^-Doped CdSiO_3_. J. Solid State Chem..

[60] Rodrigues L.C.V., Brito H.F., Hölsä J., Stefani R., Felinto M.C.F.C., Lastusaari M., Laamanen T., Nunes L.A.O. (2012). Discovery of the Persistent Luminescence Mechanism of CdSiO_3_:Tb^3+^. J. Phys. Chem. C.

[61] Dorenbos P., Vaneijk C.W.E., Bos A.J.J., Melcher C.L. (1994). Afterglow and Thermoluminescence Properties of Lu_2_SiO_5_:Ce Scintillation Crystals. J. Phys. Condens. Matter.

[62] Yamaga M., Ohsumi Y., Nakayama T., Han T.P.J. (2012). Persistent Phosphorescence in Ce-Doped Lu_2_SiO_5_. Opt. Mater. Express.

[63] Lin L., Shi C., Wang Z., Zhang W., Yin M. (2008). A Kinetics Model of Red Long-Lasting Phosphorescence in MgSiO_3_:Eu^2+^, Dy^3+^, Mn^2+^. J. Alloys Compd..

[64] Lin L., Yin M., Shi C., Zhang W. (2008). Luminescence Properties of A New Red Long-Lasting Phosphor:Mg_2_SiO_4_:Dy^3+^, Mn^2+^. J. Alloys Compd..

[65] Kuang J., Liu Y., Zhang J. (2006). White-Light-Emitting Long-Lasting Phosphorescence in Dy^3+^-Doped SrSiO_3_. J. Solid State Chem..

[66] Kuang J., Liu Y. (2005). White-Emitting Long-Lasting Phosphor Sr_2_SiO_4_:Dy^3+^. Chem. Lett..

[67] Lin H., Xu A.X., Chen G.L., Zheng Z.S., Lin H., Chen B.S., Huang L.F., Guo H.X., Xu Y. (2012). Synthesis of a New Red Long Persistent Phosphor Sr_2_ZnSi_2_O_7_:Eu^3+^, Lu^3+^ via Sol-Gel Method and Investigation of Its Luminescence. Adv. Mater. Res..

[68] Avouris P., Morgan T.N. (1981). A Tunneling Model for the Decay of Luminescence in Inorganic Phosphors: The Case of Zn_2_SiO_4_:Mn. J. Chem. Phys..

[69] Garlick G.F.J., Gibson A.F. (1948). The Electron Trap Mechanism of Luminescence in Sulphide and Silicate Phosphors. Proc. Phys. Soc..

[70] Iwasaki K., Takahashi Y., Masai H., Fujiwara T. (2009). Blue Photoluminescence, Greenish-Blue Afterglow and Their Ti-Concentration Dependence in Rare Earth-Free Bazirite-Type BaZr_1−*x*_Ti*_x_*Si_3_O_9_. Opt. Express.

[71] Takahashi Y., Masai H., Fujiwara T., Kitamura K., Inoue S. (2008). Afterglow in Synthetic Bazirite, BaZrSi_3_O_9_. J. Ceram. Soc. Jpn..

[72] Bessière A., Jacquart S., Priolkar K., Lecointre A., Viana B., Gourier D. (2011). ZnGa_2_O_4_:Cr^3+^: A New Red Long-Lasting Phosphor with High Brightness. Opt. Express.

[73] Allix M., Chenu S., Véron E., Poumeyrol T., Kouadri-Boudjelthia E.A., Alahraché S., Porcher F., Massiot D., Fayon F. (2013). Considerable Improvement of Long-Persistent Luminescence in Germanium and Tin Substituted ZnGa_2_O_4_. Chem. Mater..

[74] Jia D., Wang X.J., van der Kolk E., Yen W.M. (2002). Site Dependent Thermoluminescence of Long Persistent Phosphorescence of BaAl_2_O_4_:Ce^3+^. Opt. Commun..

[75] Jia D., Meltzer R.S., Yen W.M., Jia W., Wang X. (2002). Green Phosphorescence of CaAl_2_O_4_:Tb^3+^, Ce^3+^ Through Persistence Energy Transfer. Appl. Phys. Lett..

[76] Jia D., Wang X.J., Jia W., Yen W.M. (2003). Persistent Energy Transfer in CaAl_2_O_4_:Tb^3+^, Ce^3+^. J. Appl. Phys..

[77] Jia D., Yen W.M. (2003). Trapping Mechanism Associated with Electron Delocalization and Tunneling of CaAl_2_O_4_:Ce^3+^, a Persistent Phosphor. J. Electrochem. Soc..

[78] Liu B., Shi C., Qi Z. (2005). Potential White-Light Long-Lasting Phosphor:Dy^3+^-Doped Aluminate. Appl. Phys. Lett..

[79] Xu X., Wang Y., Li Y., Gong Y. (2009). Energy Transfer Between Eu^2+^ and Mn^2+^ in Long-Afterglow Phosphor CaAl_2_O_4_:Eu^2+^, Nd^3+^, and Mn^2+^. J. Appl. Phys..

[80] Jia D., Wang X.J., Yen W.M. (2002). Electron Traps in Tb^3+^-Doped CaAl_2_O_4_. Chem. Phys. Lett..

[81] Jia D., Yen W.M. (2003). Enhanced V_K_^3+^ Center Afterglow in MgAl_2_O_4_ by Doping with Ce^3+^. J. Lumin..

[82] Lorincz A., Puma M., James F.J., Crawford J.H.J. (1982). Thermally Stimulated Processes Involving Defects in Y^−^ and X^−^ Irradiated Spinel (MgAl_2_O_4_). J. Appl. Phys..

[83] Nakagawa H., Ebisu K., Zhang M., Kitaura M. (2003). Luminescence Properties and Afterglow in Spinel Crystals Doped with Trivalent Tb Ions. J. Lumin..

[84] Jia D. (2006). Relocalization of Ce^3+^ 5d Electrons from Host Conduction Band. J. Lumin..

[85] Jia D., Wang X.j., Jia W., Yen W.M. (2007). Trapping Processes of 5d Electrons in Ce^3+^ Doped SrAl_2_O_4_. J. Lumin..

[86] Xu X., Wang Y., Yu X., Li Y., Gong Y. (2011). Investigation of Ce-Mn Energy Transfer in SrAl_2_O_4_: Ce^3+^, Mn^2+^. J. Am. Ceram. Soc..

[87] Yu N., Liu F., Li X., Pan Z. (2009). Near Infrared Long-Persistent Phosphorescence in SrAl_2_O_4_:Eu^2+^, Dy^3+^, Er^3+^ Phosphors Based on Persistent Energy Transfer. Appl. Phys. Lett..

[88] Teng Y., Zhou J., Ma Z., Smedskjaer M.M., Qiu J. (2011). Persistent Near Infrared Phosphorescence from Rare Earth Ions Co-Doped Strontium Aluminate Phosphors. J. Electrochem. Soc..

[89] Sharma S.K., Pitale S.S., Manzar Malik M., Dubey R.N., Qureshi M.S. (2009). Luminescence Studies on the Blue-Green Emitting Sr_4_Al_14_O_25_:Ce^3+^ Phosphor Synthesized Through Solution Combustion route. J. Lumin..

[90] Luitel H.N., Watari T., Torikai T., Yada M. (2009). Luminescent Properties of Cr^3+^ Doped Sr_4_Al_14_O_25_:Eu/Dy Blue-Green and Red Phosphor. Opt. Mater..

[91] Zhong R., Zhang J., Zhang X., Lu S., Wang X.J. (2006). Red Phosphorescence in Sr_4_Al_14_O_25_:Cr^3+^, Eu^2+^, Dy^3+^ Through Persistent Energy Transfer. Appl. Phys. Lett..

[92] Zhong R., Zhang J., Zhang X., Lu S., Wang X.J. (2006). Energy Transfer and Red Phosphorescence in Strontium Aluminates Co-Doped with Cr^3+^, Eu^2+^ and Dy^3+^. J. Lumin..

[93] Zhang S., Pang R., Li C., Su Q. (2010). Green Photoluminescence, But Blue Afterglow of Tb^3+^ Activated Sr_4_Al_14_O_25_. J. Lumin..

[94] Mu Z.F., Wang Y.H., Hu Y.H., Wu H.Y., Deng L.Y., Xie W., Fu C.J., Liao C.X. (2011). The Afterglow and Thermoluminescence Properties of Y_3_Al_5_O_12_:Ce^3+^. Acta Phys. Sin..

[95] Zhang S., Li C., Pang R., Jiang L., Shi L., Su Q. (2011). Long-Lasting Phosphorescence Study on Y_3_Al_5_O_12_ Doped with Different Concentrations of Ce^3+^. J. Rare Earths.

[96] Mu Z., Hu Y., Wang Y., Wu H., Fu C., Kang F. (2011). The Structure and Luminescence Properties of Long Afterglow Phosphor Y_3−*x*_Mn*_x_*Al_5−*x*_Si*_x_*O_12_. J. Lumin..

[97] Zhang S., Li C., Pang R., Jiang L., Shi L., Su Q. (2011). Energy Transfer and Excitation Wavelength Dependent Long-Lasting Phosphorescence in Pr^3+^ Activated Y_3_Al_5_O_12_. J. Lumin..

[98] Fu J. (2000). Orange and Red Emitting Long-Lasting Phosphors MO:Eu^3+^ (M = Ca, Sr, Ba). Electrochem. Solid-State Lett..

[99] Fu J. (2002). Orange-and violet-emitting long-lasting phosphors. J. Am. Ceram. Soc..

[100] Kuang J.Y., Liu Y.L., Zhang J.X., Yuan D.S., Huang L.H., Rong J.H. (2005). Long-Lasting Phosphorescence of Tb^3+^ Doped MO (M = Ca,Sr). Chin. J. Inorg. Chem..

[101] Lu Y.Y., Liu F., Gu Z., Pan Z. (2011). Long-Lasting Near-Infrared Persistent Luminescence from β-Ga_2_O_3_:Cr^3+^ Nanowire Assemblies. J. Lumin..

[102] Pejakovic D.A. (2010). Studies of the Phosphorescence of Polycrystalline Hafnia. J. Lumin..

[103] Wiatrowska A., Zych E., Kepinski L. (2010). Monoclinic HfO_2_:Eu X-Ray Phosphor. Radiat. Meas..

[104] Zych E., Trojan-Piegza J. (2007). Anomalous Activity of Eu^3+^ in S_6_ Site of Lu_2_O_3_ in Persistent Luminescence. J. Lumin..

[105] Chen S., Yang Y., Zhou G., Wu Y., Liu P., Zhang F., Wang S., Trojan-Piegza J., Zych E. (2012). Characterization of Afterglow-Related Spectroscopic Effects in Vacuum Sintered Tb^3+^, Sr^2+^ Co-Doped Lu_2_O_3_ Ceramics. Opt. Mater..

[106] Trojan-Piegza J., Niittykoski J., Hölsä J., Zych E. (2008). Thermoluminescence and Kinetics of Persistent Luminescence of Vacuum-Sintered Tb^3+^-Doped and Tb^3+^, Ca^2+^-Codoped Lu_2_O_3_ Materials. Chem. Mater..

[107] Zych E., Trojan-Piegza J., Hreniak D., Strek W. (2003). Properties of Tb-Doped Vacuum-Sintered Lu_2_O_3_ Storage Phosphor. J. Appl. Phys..

[108] Trojan-Piegza J., Zych E., Hölsä J., Niittykoski J. (2009). Spectroscopic Properties of Persistent Luminescence Phosphors: Lu_2_O_3_:Tb^3+^, M^2+^ (M = Ca, Sr, Ba). J. Phys. Chem. C.

[109] Zhang J., Ma X., Qin Q., Shi L., Sun J., Zhou M., Liu B., Wang Y. (2012). The Synthesis and Sfterglow Luminescence Properties of a Novel Red Afterglow Phosphor:SnO_2_:Sm^3+^, Zr^4+^. Mater. Chem. Phys..

[110] Lin Y., Nan C.W., Cai N., Zhou X., Wang H., Chen D. (2003). Anomalous Afterglow from Y_2_O_3_-Based Phosphor. J. Alloys Compd..

[111] Xie W., Wang Y.H., Hu Y.H., Luo L., Wu H.Y., Deng L.Y. (2010). Preparation and Red Long-Afterglow Luminescence of Y_2_O_3_:Eu, Dy. Acta Phys. Sin..

[112] Zhang J., Pan F., Hao W., Wang T. (2006). Effect of MgO Doping on the Luminescent Properties of ZnO. Mater. Sci. Eng. B.

[113] Zhang J., Zhang Z., Wang T. (2004). A New Luminescent Phenomenon of ZnO Due to the Precipitate Trapping Effect of MgO. Chem. Mater..

[114] Zhao Z., Wang Y. (2012). The Synthesis and Afterglow Luminescence Properties of a Novel Red Afterglow Phosphor:ZrO_2_:Sm^3+^, Sn^4+^. J. Lumin..

[115] Carvalho J.M., Rodrigues L.C.V., Hölsä J., Lastusaari M., Nunes L.A.O., Felinto M.C.F.C., Malta O.L., Brito H.F. (2012). Influence of Titanium and Lutetium on the Persistent Luminescence of ZrO_2_. Opt. Mater. Express.

[116] Cong Y., Li B., Lei B., Li W. (2007). Long Lasting Phosphorescent Properties of Ti Doped ZrO_2_. J. Lumin..

[117] Cong Y., Li B., Wang X.J., Lei B., Li W. (2008). Synthesis and Optical Property Studies of Nanocrystalline ZrO_2_:Ti Long-Lasting Phosphors. J. Electrochem. Soc..

[118] Liu Y.H., Li B., Cong Y. (2010). Synthesis and Optical Property Studies of Long-Lasting Phosphor ZrO_2_:Ti Electrospinning Fibers. Spectrosc. Spectr. Anal..

[119] Wang Z., Zhang J., Zheng G., Liu Y., Zhao Y. (2012). The Unusual Variations of Photoluminescence and Afterglow Properties in Monoclinic ZrO_2_ by Annealing. J. Lumin..

[120] Xu X., Wang Y., Zeng W., Gong Y. (2011). Luminescence and Storage Properties of Sm-Doped Alkaline-Earth Atannates. J. Electrochem. Soc..

[121] Zhang J., Hu R., Qin Q., Wang D., Liu B., Wen Y., Zhou M., Wang Y. (2012). The Origin of Two Quenching Concentrations and Unusual Afterglow Behaviors of Ba_2_SnO_4_:Sm^3+^ Phosphor. J. Lumin..

[122] Gao X., Zhang Z., Wang C., Xu J., Ju Z., An Y., Liu W. (2011). The Persistent Energy Transfer and Effect of Oxygen Vacancies on Red Long-Persistent Phosphorescence Phosphors Ca_2_SnO_4_:Gd^3+^, Eu^3+^. J. Electrochem. Soc..

[123] Lei B.F., Man S.Q., Liu Y.L., Yue S. (2010). Luminescence Properties of Ca_2_SnO_4_:Eu^3+^ Red-Light Emitting Afterglow Phosphor. Chin. J. Inorg. Chem..

[124] Ju Z.H., Wei R.P., Zheng J.R., Gao X.P., Zhang S.H., Liu W.S. (2011). Synthesis and Phosphorescence Mechanism of a Reddish Orange Emissive Long Afterglow Phosphor Sm^3+^-Doped Ca_2_SnO_4_. Appl. Phys. Lett..

[125] Ju Z.H., Zhang S.H., Gao X.P., Tang X.L., Liu W.S. (2011). Reddish Orange Long Afterglow Phosphor Ca_2_SnO_4_:Sm^3+^ Prepared by Sol-Gel Method. J. Alloys Compd..

[126] Lei B., Zhang H., Mai W., Yue S., Liu Y., Man S.Q. (2011). Luminescent Properties of Orange-Emitting Long-Lasting Phosphorescence Phosphor Ca_2_SnO_4_:Sm^3+^. Solid State Sci..

[127] Jin Y., Hu Y., Chen L., Wang X., Ju G., Mu Z. (2013). Luminescent Properties of Tb^3+^-Doped Ca_2_SnO_4_ Phosphor. J. Lumin..

[128] Jiachi Z., Minghui Y., Qingsong Q., Hongliang Z., Meijiao Z., Xuhui X., Yuhua W. (2010). The Persistent Luminescence and Up Conversion Photostimulated Luminescence Properties of Nondoped Mg_2_SnO_4_ Material. J. Appl. Phys..

[129] Zhang J., Qin Q., Yu M., Zhou M., Wang Y. (2012). The photoluminescence, Afterglow and Up Conversion Photostimulated Luminescence of Eu^3+^ Doped Mg_2_SnO_4_ Phosphors. J. Lumin..

[130] Zhang J.C., Qin Q.S., Yu M.H., Zhou H.L., Zhou M.J. (2011). Photoluminescence and Persistent Luminescence Properties of Non-Doped and Ti^4+^-Doped Mg_2_SnO_4_ Phosphors. Chin. Phys. B.

[131] Lei B., Li B., Wang X., Li W. (2006). Green Emitting Long Lasting Phosphorescence (LLP) Properties of Mg_2_SnO_4_:Mn^2+^ Phosphor. J. Lumin..

[132] Wang Z.L., Zheng G.S., Wang S.Q., Qin Q.S., Zhou H.L., Zhang J.C. (2012). The Luminescence Properties of a Novel Electron Trapped Material Sr_2_SnO_4_:Sb^3+^ for Optical Storage. Acta Phys. Sin..

[133] Lei B.F., Yue S., Zhang Y.Z., Liu Y.L. (2010). Luminescence Properties of Sr_2_SnO_4_:Sm^3+^ Afterglow Phosphor. Chin. Phys. Lett..

[134] Xu X., Wang Y., Gong Y., Zeng W., Li Y. (2010). Effect of Oxygen Vacancies on the Red Phosphorescence of Sr_2_SnO_4_:Sm^3+^ Phosphor. Opt. Express..

[135] Yu X., Xu X., Qiu J. (2011). Enhanced Long Persistence of Sr_2_SnO_4_:Sm^3+^ Red Phosphor by Co-Doping with Dy^3+^. Mater. Res. Bull..

[136] Qin Q.S., Ma X.L., Shao Y., Yang X.Y., Sheng H.F., Yang J.Z., Yin Y., Zhang J.C. (2012). Synthesis and Infrared Up-Conversion Photostimulated Luminescence Properties of a Novel Optical Storage Material Sr_2_SnO_4_:Tb^3+^, Li^+^. Acta Phys. Sin..

[137] Lei B., Li B., Zhang H., Zhang L., Cong Y., Li W. (2007). Synthesis and Luminescence Properties of Cube-Structured CaSnO_3_/RE^3+^ (RE = Pr and Tb) Long-Lasting Phosphors. J. Electrochem. Soc..

[138] Lei B., Li B., Zhang H., Li W. (2007). Preparation and Luminescence Properties of CaSnO_3_:Sm^3+^ Phosphor Emitting in the Reddish Orange Region. Opt. Mater..

[139] Liang Z., Zhang J., Sun J., Li X., Cheng L., Zhong H., Fu S., Tian Y., Chen B. (2013). Enhancement of Green Long Lasting Phosphorescence in CaSnO_3_:Tb^3+^ by Addition of Alkali Ions. Phys. B Condens. Matter.

[140] Liu Z., Liu Y. (2005). Synthesis and Luminescent Properties of a New Green Afterglow Phosphor CaSnO_3_:Tb. Mater. Chem. Phys..

[141] Lei B., Man S.Q., Liu Y., Yue S. (2010). Luminescence Properties of Sm^3+^-Doped Sr_3_Sn_2_O_7_ Phosphor. Mater. Chem. Phys..

[142] Bessière A., Benhamou R.A., Wallez G., Lecointre A., Viana B. (2012). Site Occupancy and Mechanisms of Thermally Stimulated Luminescence in Ca_9_Ln(PO_4_)_7_ (Ln = lanthanide). Acta Mater..

[143] Bessière A., Lecointre A., Benhamou R.A., Suard E., Wallez G., Viana B. (2013). How to Induce Red Persistent Luminescence in Biocompatible Ca_3_(PO_4_)_2_. J. Mater. Chem. C.

[144] Lecointre A., Ait benhamou R., Bessiére A., Wallez G., Elaatmani M., Viana B. (2011). Red Long-Lasting Phosphorescence (LLP) in β-TCP type Ca_9.5_Mn(PO_4_)_7_ Compounds. Opt. Mater..

[145] Wang X., Du F., Wei D., Huang Y., Seo H.J. (2011). A New Long-Lasting Phosphor Zr^4+^ and Eu^3+^ Co-Doped SrMg_2_(PO_4_)_2_. Sens. Actuators B Chem..

[146] Jeong J., Jayasimhadri M., Lee H.S., Jang K., Yi S.S., Jeong J.H., Kim C. (2009). Photoluminescence and Phosphorescence Properties of Sr_1−*x*_Zn_2−*y*_(PO_4_)_2_:Eu^2+*x*^, Mn^2+*y*^ Phosphor for UV-Based White-LEDs. Phys. B Condens. Matter.

[147] Peng Z., Xu Z., Luo C., Yu J., Zhang G. (2008). Synthesis and Luminescent Properties of a Novel Bluish-White Afterglow Phosphor, *b-*Zn_3_(PO_4_)_2_:Hf^4+^. Lumin..

[148] Song Y.H., Zou H.F., Gan S.C., Deng Y.F., Hong G.Y., Meng J. (2007). Phase Conversion and Spectral Properties of Long Lasting Phosphor Zn_3_(PO_4_)_2_:Mn^2+^, Ga^3+^. J. Mater. Sci..

[149] Wang J., Su Q., Wang S.B. (2005). A Novel Red Long Lasting Phosphorescent (LLP) Material β-Zn_3_(PO_4_)_2_:Mn^2+^, Sm^3+^. Mater. Res. Bull..

[150] Wang J., Wang S., Su Q. (2004). The Role of Excess Zn^2+^ Ions in Improvement of Red Long Lasting Phosphorescence (LLP) Performance of β-Zn_3_(PO_4_)_2_:Mn Phosphor. J. Solid State Chem..

[151] Wang J., Wang S.B., Su Q. (2004). Synthesis, Photoluminescence and Thermostimulated-Luminescence Properties of Novel Red Long-Lasting Phosphorescent Materials β-Zn_3_(PO_4_)_2_:Mn^2+^, M^3+^ (M = Al and Ga). J. Mater. Chem..

[152] Wang J., Su Q., Wang S.B. (2005). Blue and Red Long Lasting Phosphorescence (LLP) in β-Zn_3_(PO_4_)_2_:Mn^2+^, Zr^4+^. J. Phys. Chem. Solids.

[153] Lecointre A., Bessière A., Bos A.J.J., Dorenbos P., Viana B., Jacquart S. (2011). Designing a Red Persistent Luminescence Phosphor: The Example of YPO_4_:Pr^3+^, Ln^3+^ (Ln = Nd, Er, Ho, Dy). J. Phys. Chem. C.

[154] Zhang X.Y., Cheng G., Mi X.Y., Xiao Z.Y., Jiang W.W., Hu J.J. (2004). Preparation and Long Persistence Red Luminescence of M_0.2_Ca_0.8_TiO_3_:Pr^3+^ (M = Mg^2+^, Sr^2+^, Ba^2+^, Zn^2+^). J. Rare Earths.

[155] Boutinaud P., Sarakha L., Cavalli E., Bettinelli M., Dorenbos P., Mahiou R. (2009). About Red Afterglow in Pr^3+^ Doped Titanate Perovskites. J. Phys. D Appl. Phys..

[156] Jia W., Jia D., Rodriguez T., Evans D.R., Meltzer R.S., Yen W.M. (2006). UV Excitation and Trapping Centers in CaTiO_3_:Pr^3+^. J. Lumin..

[157] Pan Y.X., Su Q., Xu H.F., Chen T.H., Ge W.K., Yang C.L., Wu M.M. (2003). Synthesis and Red Luminescence of Pr^3+^-Doped CaTiO_3_ Nanophosphor from Polymer Precursor. J. Solid State Chem..

[158] Zhang X., Zhang J., Nie Z., Wang M., Ren X., Wang X.j. (2007). Enhanced Red Phosphorescence in Nanosized CaTiO_3_:Pr^3+^ Phosphors. Appl. Phys. Lett..

[159] Zhang X., Zhang J., Zhang X., Chen L., Lu S., Wang X.J. (2007). Enhancement of Red Fluorescence and Afterglow in CaTiO_3_:Pr^3+^ by Addition of Lu_2_O_3_. J. Lumin..

[160] Haranath D., Khan A.F., Chander H. (2006). Bright Red Luminescence and Energy Transfer of Pr^3+^-Doped (Ca, Zn) TiO_3_ Phosphor for Long Decay Applications. J. Phys. D Appl. Phys..

[161] Wanjun T., Donghua C. (2007). Photoluminescent Properties of (Ca,Zn)TiO_3_:Pr, B Particles Synthesized by the Peroxide-Based Route Method. J. Am. Ceram. Soc..

[162] Yuan X., Shi X., Shen M., Wang W., Fang L., Zheng F., Wu X. (2009). Luminescent Properties of Pr^3+^ Doped (Ca, Zn)TiO_3_: Powders and Films. J. Alloys Compd..

[163] Lian S.X., Qi Y., Rong C.Y., Yu L.P., Zhu A.L., Yin D.L., Liu S.B. (2010). Effectively Leveraging Solar Energy Through Persistent Dual Red Phosphorescence: Preparation, Characterization, and Density Functional Theory Study of Ca_2_Zn_4_Ti_16_O_38_:Pr^3+^. J. Phys. Chem. C.

[164] Qi Y., Lian S.X., Yu L.P., Zhou W., Yin D.L. (2009). Synthesis and Red Persistent Properties of Phosphor Ca_2_Zn_4_Ti_16_O_38_:Pr^3+^, Na^+^. Chin. J. Inorg. Chem..

[165] Chu M.H., Jiang D.P., Zhao C.J., Li B. (2010). Long-Lasting Phosphorescence Properties of Pyrochlore La_2_Ti_2_O_7_:Pr^3+^ Phosphor. Chin. Phys. Lett..

[166] Blasse G., Grabmaier B.C., Ostertag M. (1993). The Afterglow Mechanism of Chromium-Doped Gadolinium Gallium garnet. J. Alloys Compd..

[167] Kostyk L., Luchechko A., Zakharko Y., Tsvetkova O., Kukliński B. (2009). Cr-Related Centers in Gd_3_Ga_5_O_12_ Polycrystals. J. Lumin..

[168] Matsui H., Xu C.N., Akiyama M., Watanabe T. (2000). Strong Mechanoluminescence from UV-Irradiated Spinels of ZnGa_2_O_4_:Mn and MgGa_2_O_4_:Mn. Jpn. J. Appl. Phys..

[169] Zhuang Y., Ueda J., Tanabe S. (2012). Photochromism and White Long-Lasting Persistent Luminescence in Bi^3+^-Doped ZnGa_2_O_4_ Ceramics. Opt. Mater. Express.

[170] Uheda K., Maruyama T., Takizawa H., Endo T. (1997). Synthesis and Long-Period Phosphorescence of ZnGa_2_O_4_:Mn^2+^ Spinel. J. Alloys Compd..

[171] Che G., Li X., Liu C., Wang H., Liu Y., Xu Z. (2008). Long-Lasting Phosphorescence Properties of Mn^2+^-Doped Cd_2_Ge_7_O_16_ Orange Light-Emitting Phosphor. Physica Status Solidi A.

[172] Yi S.J., Liu Y.L., Zhang J.X., Yuan D.S. (2004). Long Phosphorescence Persistence Property of Cd_2_Ge_7_O_16_:Pb^2+^. Chem. J. Chin. Univ..

[173] Cong Y., Li B., Yue S., Zhang L., Li W., Wang X.J. (2009). Enhanced Red Phosphorescence in MgGeO_3_:Mn^2+^ by Addition of Yb^3+^ Ions. J. Electrochem. Soc..

[174] Iwasaki M., Kim D.N., Tanaka K., Murata T., Morinaga K. (2003). Red Phosphorescence Properties of Mn Ions in MgO-GeO_2_ Compounds. Sci. Technol. Adv. Mater..

[175] Sun Z.X. (2012). Enhanced Green-Light-Emitting Afterglow in Zn_2_GeO_4_: Mn^2+^ Phosphor by Yb^3+^ Codoping. Chin. J. Inorg. Chem..

[176] Che G.B., Liu C.B., Wang Q.W., Xu Z.L. (2008). White-Light-Emission Afterglow Phosphor CaZnGe_2_O_6_:Dy^3+^. Chem. Lett..

[177] Che G., Liu C., Li X., Xu Z., Liu Y., Wang H. (2008). Luminescence Properties of a New Mn^2+^-Activated Red Long-Afterglow Phosphor. J. Phys. Chem. Solids.

[178] Liu C., Che G., Xu Z., Wang Q. (2009). Luminescence Properties of a Tb^3+^ Activated Long-Afterglow Phosphor. J. Alloys Compd..

[179] Woo B.K., Luo Z., Li Y., Singh S.P., Joly A.G., Hossu M., Liu Z., Chen W. (2011). Luminescence Enhancement of CaZnGe_2_O_6_:Tb^3+^ Afterglow Phosphors Synthesized Using ZnO Nanopowders. Opt. Mater..

[180] Liu Z., Liu Y. (2005). Afterglow Energy Transfer in Cd_3_Al_2_Ge_3_O_12_:Dy. Phys. Stat. Sol. A.

[181] Jia D., Lewis L.A., Wang X.J. (2010). Cr^3+^-Doped Lanthanum Gallogermanate Phosphors with Long Persistent IR Emission. Electrochem. Solid-State Lett..

[182] Yan W., Liu F., Lu Y.Y., Wang X.J., Yin M., Pan Z. (2010). Near Infrared Long-Persistent Phosphorescence in La_3_Ga_5_GeO_14_:Cr^3+^ Phosphor. Opt. Express.

[183] Kang F.W., Hu Y.H., Wu H.Y., Ju G.F. (2011). Red Afterglow Properties of Eu^3+^ in CaMoO_4_ Phosphor. Chin. Phys. Lett..

[184] Boutinaud P., Sarakha L., Mahiou R. (2009). NaNbO_3_:Pr^3+^: A New Red Phosphor Showing Persistent Luminescence. J. Phys. Condens. Matter.

[185] Takayama T., Katsumata T., Komuro S., Morikawa T. (2005). Growth and Characteristics of a New Long Afterglow Phosphorescent Yttrium Tantalate Crystal. J. Cryst. Growth.

[186] Wu H., Hu Y., Kang F., Li N., Ju G., Mu Z., Yang Z. (2012). Luminescent Properties of Praseodymium in CaWO_4_ Matrix. J. Am. Ceram. Soc..

[187] Kang F., Hu Y., Chen L., Wang X., Mu Z., Wu H., Ju G. (2012). Eu^3+^ Doped CaWO_4_: A Potential Red Long Afterglow Phosphor. Appl. Phys. B.

[188] Liu Z.W., Liu Y.L., Yuan D.S., Zhang J.X., Rong J.H., Huang L.H. (2004). Long-Lasting Phosphorescence in Eu^3+^-Doped CaWO_4_. Chin. J. Inorg. Chem..

[189] Wu H.Y., Hu Y.H., Kang F.W., Li N.N. (2012). Enhancement on Afterglow Properties of Eu^3+^ by Ti^4+^, Mg^2+^ Incorporation in CaWO_4_ Matrix. J. Mater. Res..

[190] Kang F., Hu Y., Wu H., Mu Z., Ju G., Fu C., Li N. (2012). Luminescence and Red Long Afterglow Investigation of Eu^3+^-Sm^3+^ Co-Doped CaWO_4_ Phosphor. J. Lumin..

[191] Wu H., Hu Y., Kang F., Chen L., Wang X., Ju G., Mu Z. (2011). Observation on Long Afterglow of Tb^3+^ in CaWO_4_. Mater. Res. Bull..

[192] Moon C., Nishi M., Miura K., Hirao K. (2009). Blue Long-Lasting Phosphorescence of Ti-Doped BaZrO_3_ Perovskites. J. Lumin..

[193] Sun D., Li D., Zhu Z., Xiao J., Tao Z., Liu W. (2012). Photoluminescence Properties of Europium and Titanium Co-Doped BaZrO_3_ Phosphors Powders Synthesized by the Solid-State Reaction Method. Opt. Mater..

[194] Harvey E.N. (1957). A History of Luminescence from the Earliest Times Until 1900.

[195] Lastusaari M., Laamanen T., Malkamäki M., Eskola K.O., Kotlov A., Carlson S., Welter E., Brito H.F., Bettinelli M., Jungner H., Hölsä J. (2012). The Bologna Stone: History’s First Persistent Luminescent Material. Eur. J. Mineral..

[196] Poelman D., Avci N., Smet P.F. (2009). Measured Luminance and Visual Appearance of Multi-Color Persistent Phosphors. Opt. Express.

[197] Garlick G.F.J., Mason D.E. (1949). Electron Traps and Infrared Stimulation of Phosphors. J. Electrochem. Soc..

[198] Lawangar R.D., Shalgaonkar C.S., Pawar S.H., Narlikar A.V. (1972). Thermally Stimulated Luminescence of CaS: Bi, Pd Phosphors. Solid State Commun..

[199] Pawar S.H., Narlikar A.V. (1976). Mechanism of Luminescence in CaS: Bi Phosphor. Mater. Res. Bull..

[200] Jia D., Meltzer R.S., Yen W.M. (2002). Ce^3+^ Energy Levels Relative to the Band Structure in CaS: Evidence from Photoionization and Electron Trapping. J. Lumin..

[201] Paulose P.I., Joseph J., Rudra Warrier M.K., Jose G., Unnikrishnan N.V. (2007). Relaxation Kinetics of Sm:Ce-Doped CaS Phosphors. J. Lumin..

[202] Jia D., Zhu J., Wu B. (2000). Improvement of Persistent Phosphorescence of Ca_0.9_Sr_0.1_S:Bi^3+^ by Codoping Tm^3+^. J. Lumin..

[203] Pitale S.S., Sharma S.K., Dubey R.N., Qureshi M.S., Malik M.M. (2008). TL and PL Studies on Defect-Assisted Green Luminescence from Doped Strontium Sulfide Phosphor. J. Lumin..

[204] Clabau F., Rocquefelte X., Le Mercier T., Deniard P., Jobic S., Whangbo M.H. (2006). Formulation of Phosphorescence Mechanisms in Inorganic Solids Based on a New Model of Defect Conglomeration. Chem. Mater..

[205] Ma L., Chen W. (2011). Enhancement of Afterglow in ZnS: Cu, Co Water-Soluble Nanoparticles by Aging. J. Phys. Chem. C.

[206] Lei B., Liu Y., Zhang J., Meng J., Man S., Tan S. (2010). Persistent Luminescence in Rare Earth Ion-Doped Gadolinium Oxysulfide Phosphors. J. Alloys Compd..

[207] Zhang J.W., Liu Y.L., Zhang J.X., Yuan D.S., Rong J.H., Huang L.H. (2006). Long Afterglow Property and Mechanism on Gd_2_O_2_S:Ti. Rare Metal. Mater. Eng..

[208] Zhang J., Liu Y.L., Man S.Q. (2006). Afterglow Phenomenon in Erbium and Titanium Codoped Gd_2_O_2_S Phosphors. J. Lumin..

[209] Hang T., Liu Q., Mao D., Chang C. (2008). Long Lasting Behavior of Gd_2_O_2_S:Eu^3+^ Phosphor Synthesized by Hydrothermal Routine. Mater. Chem. Phys..

[210] Mao S., Liu Q., Gu M., Mao D., Chang C. (2008). Long Lasting Phosphorescence of Gd_2_O_2_S:Eu, Ti, Mg Nanorods via a Hydrothermal Routine. J. Alloys Compd..

[211] Liu G., Zhang Q., Wang H., Li Y. (2012). A Reddish La_2_O_2_S-Based Long-Afterglow Phosphor with Effective Absorption in the Visible Light Region. Mater. Sci. Eng. B.

[212] Kang C.C., Liu R.S., Chang J.C., Lee B.J. (2003). Synthesis and Luminescent Properties of a New Yellowish-Orange Afterglow Phosphor Y_2_O_2_S:Ti, Mg. Chem. Mater..

[213] Liu C.B., Che G.B. (2006). Observation of Enhanced Long-Lasting Phosphorescence in Y_2_O_2_S:RE^3+^ (RE = Lu, Gd) Phosphors. Physica Status Solidi A.

[214] Wang L., Zhang L., Huang Y., Jia D., Lu J. (2009). Effects of Gd^3+^ and Lu^3+^ Co-Doping on the Long Afterglow Properties of Yellowish-Orange Phosphor Y_2_O_2_S:Ti^4+^, Mg^2+^. J. Lumin..

[215] Zhang P., Hong Z., Wang M., Fang X., Qian G., Wang Z. (2005). Luminescence Characterization of a New Long Afterglow Phosphor of Single Ti-Doped Y_2_O_2_S. J. Lumin..

[216] Zhang P.Y., Wang M.Q., Hong Z.L., Fang X.P., Qian G.D., Wang Z.Y. (2004). A New Yellow Long Lasting Phosphor Y_2_O_2_S:Ti. J. Rare Earths.

[217] Wang X., Zhang Z., Tang Z., Lin Y. (2003). Characterization and Properties of a Red and Orange Y_2_O_2_S-Based Long Afterglow Phosphor. Mater. Chem. Phys..

[218] Wang Y.H., Wang Z.L. (2006). Characterization of Y_2_O_2_S:Eu^3+^, Mg^2+^, Ti^4+^ Long-Lasting Phosphor Synthesized by Flux Method. J. Rare Earths.

[219] Yuan S., Yang Y., Fang B., Chen G. (2007). Effects of Doping Ions on Afterglow Properties of Y_2_O_2_S:Eu Phosphors. Opt. Mater..

[220] Zhang J.Y., Zhang Z.T., Tang Z.L., Wang T.M. (2004). A New Method to Synthesize Long Afterglow Red Phosphor. Ceram. Int..

[221] Lei B., Liu Y., Tang G., Ye Z., Shi C. (2004). Spectra and Long-Lasting Properties of Sm^3+^-Doped Yttrium Oxysulfide Phosphor. Mater. Chem. Phys..

[222] Lei B.F., Liu Y.L., Tang G.B., Ye Z.R., Shi C.S. (2003). A New Orange-Red Long-Lasting Phosphor Material Y_2_O_2_S:Sm^3+^. Chem. J. Chin. Univ..

[223] Yao K., Wang M., Liu S., Zhang L., Li W. (2006). Effects of Host Doping on Spectral and Long-Lasting Properties of Sm^3+^-Doped Y_2_O_2_S. J. Rare Earths.

[224] Liu B., Shi C., Qi Z. (2006). White-Light Long-Lasting Phosphorescence from Tb^3+^-Activated Y_2_O_2_S Phosphor. J. Phys. Chem. Solids.

[225] Hölsä J., Laamanen T., Lastusaari M., Malkamäki M., Niittykoski J., Zych E. (2009). Effect of Mg^2+^ and Ti^IV^ Doping on the Luminescence of Y_2_O_2_S:Eu^3+^. Opt. Mater..

[226] Hong Z., Zhang P., Fan X., Wang M. (2007). Eu^3+^ Red Long Afterglow in Y_2_O_2_S:Ti, Eu Phosphor Through Afterglow Energy Transfer. J. Lumin..

[227] Lei B.F., Liu Y.L., Tang G.B., Ye Z.R., Shi C.S. (2003). Unusual Afterglow Properties of Tm^3+^ Doped Yttrium Oxysulfide. Chem. J. Chin. Univ..

[228] Liu X., Qiao Y., Dong G., Ye S., Zhu B., Zhuang Y., Qiu J. (2009). BCNO-Based Long-Persistent Phosphor. J. Electrochem. Soc..

[229] Wang W.N., Ogi T., Kaihatsu Y., Iskandar F., Okuyama K. (2011). Novel Rare-Earth-Free Tunable-Color-Emitting BCNO Phosphors. J. Mater. Chem..

[230] Ju G., Hu Y., Chen L., Wang X. (2012). Persistent Luminescence and Its Mechanism of Ba_5_(PO_4_)_3_Cl:Ce^3+^, Eu^2+^. J. Appl. Phys..

[231] Zhang J.S., Zhong H.Y., Sun J.S., Cheng L.H., Li X.P., Chen B.J. (2012). Reddish Orange Long-Lasting Phosphorescence in KY_3_F_10_:Sm^3+^ for X-Ray or Cathode Ray Tubes. Chin. Phys. Lett..

[232] Uheda K., Takizawa H., Endo T., Miura C., Shimomura Y., Kijima N., Shimada M. (2001). Photo- and Thermo-Luminescence of Zinc Silicon Nitride Doped with Divalent Manganese. J. Mater. Sci. Lett..

[233] Qiu J., Miura K., Inouye H., Kondo Y., Mitsuyu T., Hirao K. (1998). Femtosecond Laser-Induced Three-Dimensional Bright and Long-Lasting Phosphorescence Inside Calcium Aluminosilicate Glasses Doped with Rare Earth Ions. Appl. Phys. Lett..

[234] Kinoshita T., Hosono H. (2000). Materials Design and Example of Long Lasting Phosphorescent Glasses Utilizing Electron Trapped Centers. J. Non-Cryst. Solids.

[235] Hosono H., Kinoshita T., Kawazoe H., Yamazaki M., Yamamoto Y., Sawanobori N. (1998). Long Lasting Phosphorescence Properties of Tb^3+^-Activated Reduced Calcium Aluminate Glasses. J. Phys. Condens. Matter.

[236] Kinoshita T., Yamazaki M. (1999). Long Lasting Phosphorescence and Photostimulated Luminescence in Tb-Ion-Activated Reduced Calcium. J. Appl. Phys..

[237] Qiu J., Wada N., Ogura F., Kojima K., Hirao K. (2002). Structural Relaxation and Long-Lasting Phosphorescence in Sol-Gel-Derived GeO_2_ Glass After Ultraviolet Light Irradiation. J. Phys. Condens. Matter.

[238] Wada N., Ogura F., Yamamoto K., Kojima K. (2005). White Luminescence and Afterglow in Germanium Oxide Glasses Prepared by the Sol-Gel Method. Glass Technol..

[239] Qiu J., Gaeta A.L., Hirao K. (2001). Long-Lasting Phosphorescence in Oxygen-Deficient Ge-Doped Silica Glasses at Room Temperature. Chem. Phys. Lett..

[240] Qiu J., Kondo Y., Miura K., Mitsuyu T., Hirao K. (1999). Infrared Femtosecond Laser Induced Visible Long-Lasting Phosphorescence in Mn^2+^-Doped Sodium Borate Glasses. Jpn. J. Appl. Phys..

[241] Yamazaki M., Kojima K. (2004). Long-Lasting Afterglow in Tb^3+^-Doped SiO_2_-Ga_2_O_3_-CaO-Na_2_O Glasses and Its Sensitization by Yb^3+^. Solid State Commun..

[242] Qiu J., Miyauchi K., Kawamoto Y., Kitamura N., Qiu J., Hirao K. (2002). Long-Lasting Phosphorescence in Sn^2+^-Cu^2+^ Codoped Silicate Glass and Its High-Pressure Treatment Effect. Appl. Phys. Lett..

[243] Zhang L., Li C., Su Q. (2006). Long Lasting Phosphorescence in Eu^2+^ and Ce^3+^ Co-Doped Strontium Borate Glasses. J. Rare Earths.

[244] Sanada T., Seto H., Morimoto Y., Yamamoto K., Wada N., Kojima K. (2010). Luminescence and Long-Lasting Afterglow in Mn^2+^ and Eu^3+^ Co-Doped ZnO–GeO_2_ Glasses and Glass Ceramics Prepared by Sol-Gel Method. J. Sol-Gel Sci. Technol..

[245] Takahashi Y., Ando M., Ihara R., Fujiwara T. (2011). Green-Emissive Mn-Activated Nanocrystallized Glass with Willemite-Type Zn_2_GeO_4_. Opt. Mater. Express.

[246] Jiang X.W., Qiu J.R., Zeng H.D., Zhu C.S. (2003). Femtosecond Laser-Induced Long-Lasting Phosphorescence in Pr^3+^-Doped ZnO-B_2_O_3_-SiO_2_ Glass. Chin. Phys..

[247] Wang Z.Y., Zhang F.A., Guo X.R., Wang Y.H., Fan X.P., Qian G.D. (2006). Study on Long-Lasting Phosphorescent Mechanism of Tb^3+^ Doped ZnO-B_2_O_3_-SiO_2_ Glass. J. Zhejiang Univ..

[248] Yamazaki M., Yamamoto Y., Nagahama S., Sawanobori N., Mizuguchi M., Hosono H. (1998). Long Luminescent Glass:Tb^3+^-Activated ZnO-B_2_O-SiO_2_ Glass. J. Non-Cryst. Solids.

[249] Li C., Su Q., Wang S. (2002). Multi-Color Long-Lasting Phosphorescence in Mn^2+^-Doped ZnO-B_2_O_3_-SiO_2_ Glass-Ceramics. Mater. Res. Bull..

[250] Li C., Yu Y., Wang S., Su Q. (2003). Photo-Stimulated Long-Lasting Phosphorescence in Mn^2+^-Doped Zinc Borosilicate Glasses. J. Non-Cryst. Solids.

[251] Li C., Su Q. (2004). Action of Co-Dopant in Electron-Trapping Materials: The Case of Sm^3+^ in Mn^2+^-Activated Zinc Borosilicate Glasses. Appl. Phys. Lett..

[252] Li C., Su Q. (2006). Effect of Samarium on Mn Activated Zinc Borosilicate Storage Glasses. J. Rare Earths.

[253] Li C., Wang J., Liang H., Su Q. (2007). Near Infrared Long Lasting Emission of Yb^3+^ and Its Influence on the Optical Storage Ability of Mn^2+^-Activated Zinc Borosilicate Glasses. J. Appl. Phys..

[254] Lin G., Dong G., Tan D., Liu X., Zhang Q., Chen D., Qiu J., Zhao Q., Xu Z. (2010). Long Lasting Phosphorescence in Oxygen-Deficient Zinc-Boron-Germanosilicate Glass-Ceramics. J. Alloys Compd..

[255] Smet P.F., Van den Eeckhout K., Bos A.J.J., van der Kolk E., Dorenbos P. (2012). Temperature and Wavelength Dependent Trap Filling in M_2_Si_5_N_8_:Eu (M = Ca, Sr, Ba) Persistent Phosphors. J. Lumin..

[256] Korthout K., Van den Eeckhout K., Botterman J., Nikitenko S., Poelman D., Smet P.F. (2011). Luminescence and X-Ray Absorption Measurements of Persistent SrAl_2_O_4_: Eu, Dy Powders: Evidence for Valence State Changes. Phys. Rev. B.

[257] Smet P.F., Parmentier A.B., Poelman D. (2011). Selecting Conversion Phosphors for White-Light Emitting Diodes. J. Electrochem. Soc..

[258] Bos A.J.J., van Duijvenvoorde R.M., van der Kolk E., Drozdowski W., Dorenbos P. (2011). Thermoluminescence Excitation Spectroscopy: A Versatile Technique to Study Persistent Luminescence Phosphors. J. Lumin..

[259] Van den Eeckhout K., Bos A.J.J., Poelman D., Smet P.F. (2013). Revealing Trap Depth Distributions in Persistent Phosphors. Phys. Rev. B.

[260] Chen R., McKeever S.W.S. (1997). Section 2.4.3, Continua; Trap Distributions. Theory of Thermoluminescence and Related Phenomena.

[261] Takeyama T., Nakamura T., Takahashi N., Ohta M. (2004). Electron Paramagnetic Resonance Studies on the Defects Formed in the Dy(III)-Doped SrAl_2_O_4_. Solid State Sci..

[262] Poelman D., Smet P.F. (2010). Photometry in the Dark: Time Dependent Visibility of Low Intensity Light Sources. Opt. Express.

[263] Poelman D., Smet P.F. (2011). Photometry in the Dark: Time Dependent Visibility of Low Intensity Light Sources: Erratum. Opt. Express.

